# Hematocrit, hemoglobin and red blood cells are associated with vascular function and vascular structure in men

**DOI:** 10.1038/s41598-020-68319-1

**Published:** 2020-07-10

**Authors:** Shinji Kishimoto, Tatsuya Maruhashi, Masato Kajikawa, Shogo Matsui, Haruki Hashimoto, Yuji Takaeko, Takahiro Harada, Takayuki Yamaji, Yiming Han, Yasuki Kihara, Kazuaki Chayama, Chikara Goto, Farina Mohamad Yusoff, Ayumu Nakashima, Yukihito Higashi

**Affiliations:** 1 Department of Cardiovascular Regeneration and Medicine, Research Institute for Radiation Biology and Medicine, Hiroshima University, 1-2-3 Kasumi, Minami-ku, Hiroshima, 734-8551 Japan; 2 Department of Cardiovascular Medicine, Graduate School of Biomedical and Health Sciences, Hiroshima University, Hiroshima, Japan; 3 Division of Regeneration and Medicine, Medical Center for Translational and Clinical Research, Hiroshima University Hospital, Hiroshima, Japan; 4 Department of Cardiovascular Medicine, Hiroshima Prefectural Hospital, Hiroshima, Japan; 5 Department of Gastroenterology and Metabolism, Graduate School of Biomedical and Health Sciences, Hiroshima University Hiroshima, Hiroshima, Japan; 6 Dpartment of Rehabilitation, Faculty of General Rehabilitation, Hiroshima International University, Hiroshima, Japan; 7 Department of Stem Cell Biology and Medicine, Graduate School of Biomedical and Health Sciences, Hiroshima University Hiroshima, Hiroshima, Japan

**Keywords:** Cardiology, Cardiovascular biology, Biomarkers

## Abstract

High and low hematocrit (Hct) and hemoglobin (Hb) levels are associated with the risk of cardiovascular disease. The purpose of this study was to determine the relationships of Hct, Hb and red blood cells (RBCs) with vascular function and structure. We measured flow-mediated vasodilation (FMD), nitroglycerin-induced vasodilation (NID), brachial intima media thickness (IMT), and brachial-ankle pulse wave velocity (baPWV) in 807 men. The subjects were divided into six groups according to the levels of Hct, Hb and RBCs. NID was highest in the 46.0–48.9% Hct group among the six groups according to Hct levels. Brachial IMT was lowest in the 46.0–48.9% Hct group among the six groups. There were no significant differences in FMD and baPWV among the six groups. We used 46.0–48.9% Hct as a reference to define the lower tertile. The adjusted odds ratio of being in the low tertile of NID was significantly higher in the < 42.9% and ≥ 49.0% Hct groups. Adjusted odds ratio of being in the low tertile of brachial IMT was significantly lower in the < 39.9% Hct groups. Similar results were obtained for Hb and RBCs. Low and high levels of Hct, Hb and RBCs were associated with vascular smooth muscle dysfunction, and low Hct levels were associated with abnormal vascular structure. Increases in the levels of Hct, Hb and RBCs within normal ranges may have beneficial effects on the vasculature.

## Introduction

Hematocrit (Hct), the volume percentage of red blood cells (RBCs) in total blood, and hemoglobin (Hb) are associated with a risk of cardiovascular disease. A high Hct level has been shown to be associated with an increased risk of cardiovascular disease^1–3^. On the other hand, J- or U-shaped relations between Hct and morbidity and mortality from cardiovascular events have been shown^1^. The relationship between a low Hct level and cardiovascular disease is controversial^1,3,4^. It is well known that Hct and Hb levels are major determinants of blood viscosity and oxygen delivery dynamics. It is thought that changes in blood viscosity and oxygen delivery dynamics alter vascular function and structure. Indeed, Lee et al. showed that high blood viscosity was associated with increased carotid intima-media thickness (IMT)^5^. However, there is no information on the associations of Hct, Hb and RBCs with vascular function and vascular structure.

Endothelial dysfunction is the initial step of atherosclerosis and leading to the development and progression of this condition^6,7^. Recently, flow-mediated vasodilation (FMD) as an index of endothelium-dependent vasodilation and nitroglycerin-induced vasodilation (NID) and an index of endothelium-independent vasodilation have been widely used as methods for assessment of endothelial function and vascular smooth muscle function, respectively^8,9^. Measurement of FMD reflects the response to the release of nitric oxide (NO). Moreover, growing evidence has shown that endothelial function assessed by FMD and vascular smooth muscle function assessed by NID can serve as independent predictors of cardiovascular events^10–12^. Measurement of brachial IMT in the artery as an index of structural change of the artery and measurement of brachial-ankle pulse wave velocity (baPWV) as an index of arterial stiffness have be shown to be significantly correlated with cardiovascular risk factors^13,14^.

The purpose of this study was to evaluate the relationships of levels of Hct, Hb and RBCs with vascular function and vascular structure and to evaluate the optimal cutoff levels of Hct, Hb and RBCs for maintenance of vascular function and vascular structure.

## Results

### Baseline clinical characteristics

The baseline clinical characteristics of the subjects are summarized in Table 1. Of the 807 subjects, 627 (77.7%) had hypertension, 496 (61.5%) had dyslipidemia, 269 (33.3%) had diabetes mellitus, 171 (21.2%) had previous coronary artery disease, 70 (8.7%) had previous stroke, and 188 (23.3%) were current smokers. Mean values were 3.5 ± 2.6% for FMD, 11.7 ± 5.8% for NID, 0.34 ± 0.08 mm for brachial IMT and 1683 ± 382 cm/s for baPWV.

**Table 1:** Clinical characteristics of the subjects according to hematocrit levels.

Variables	Total (n = 807)	Hematocrit < 37.0% (n = 111)	Hematocrit 37.0–39.9% (n = 138)	Hematocrit 40.0–42.9% (n = 221)	Hematocrit 43.0–45.9% (n = 214)	Hematocrit 46.0–48.9% (n = 91)	Hematocrit 49.0% ≤ (n = 32)	P value
Age, year	62 ± 14	71 ± 12	67 ± 11	62 ± 13	59 ± 14	55 ± 14	54 ± 12	< 0.01
Body mass index, kg/m^2^	24.7 ± 3.9	23.1 ± 3.6	24.1 ± 3.2	24.6 ± 3.5	25.1 ± 4.4	25.9 ± 3.5	26.9 ± 4.4	< 0.01
Systolic blood pressure, mmHg	134 ± 19	135 ± 21	133 ± 19	132 ± 18	134 ± 19	135 ± 20	141 ± 20	0.17
Diastolic blood pressure, mmHg	80 ± 12	75 ± 12	78 ± 11	80 ± 12	81 ± 12	83 ± 12	85 ± 12	< 0.01
Heart rate, bpm	70 ± 13	68 ± 13	70 ± 12	70 ± 13	70 ± 13	73 ± 13	72 ± 11	0.08
Total cholesterol, mmol/L	4.86 ± 0.96	4.58 ± 0.83	4.71 ± 0.88	4.84 ± 0.93	5.09 ± 0.96	5.12 ± 0.93	5.09 ± 1.16	< 0.01
Triglycerides, mmol/L	1.70 ± 1.20	1.46 ± 1.10	1.47 ± 0.98	1.64 ± 1.04	1.76 ± 1.05	2.09 ± 1.64	2.59 ± 2.07	< 0.01
HDL cholesterol, mmol/L	1.47 ± 0.44	1.50 ± 0.49	1.55 ± 0.49	1.42 ± 0.44	1.47 ± 0.39	1.37 ± 0.36	1.37 ± 0.34	0.01
LDL cholesterol, mmol/L	2.84 ± 0.85	2.46 ± 0.78	2.66 ± 0.75	2.84 ± 0.83	3.05 ± 0.91	3.05 ± 0.85	2.92 ± 0.75	< 0.01
Glucose, mmol/L	6.77 ± 2.50	6.99 ± 2.89	6.94 ± 2.22	6.72 ± 2.33	6.49 ± 2.00	6.61 ± 2.39	7.99 ± 5.11	0.05
Hemoglobin A1c, %	5.8 ± 1.0	6.1 ± 1.5	5.8 ± 0.8	5.7 ± 0.8	5.8 ± 0.8	5.9 ± 1.1	6.3 ± 1.8	0.04
BUN, mmol/L	5.71 ± 1.93	7.50 ± 2.93	6.43 ± 2.03	5.36 ± 1.39	5.36 ± 1.39	5.36 ± 1.39	5.71 ± 1.93	< 0.01
Creatinine, mmol/L	81.3 ± 25.6	100.8 ± 46.0	82.2 ± 27.4	78.7 ± 15.0	74.3 ± 17.7	76.9 ± 15.9	84.0 ± 18.6	< 0.01
eGFR, mL/min/1.73 m^2^	71 ± 20	59 ± 23	69 ± 21	71 ± 15	78 ± 19	77 ± 18	69 ± 16	< 0.01
**Medical history, n (%)**								
Hypertension	627 (77.7)	88 (79.3)	118 (85.5)	174 (78.7)	151 (70.6)	67 (73.6)	29 (90.6)	< 0.01
Dyslipidemia	496 (61.5)	69 (62.2)	82 (59.4)	135 (61.1)	129 (60.3)	58 (63.7)	23 (71.9)	0.84
Diabetes mellitus	269 (33.3)	47 (42.3)	43 (31.2)	69 (31.2)	69 (32.2)	30 (33.0)	11 (34.4)	0.42
Previous coronary heart disease	171 (21.2)	36 (32.4)	44 (31.9)	46 (20.8)	21 (9.8)	14 (15.4)	10 (31.3)	< 0.01
Previous stroke	70 (8.7)	19 (17.1)	17 (12.3)	14 (6.3)	11 (5.1)	6 (6.6)	3 (9.4)	< 0.01
Current smoker, n (%)	188 (23.3)	18 (16.2)	17 (12.3)	40 (18.1)	66 (30.8)	34 (37.4)	13 (40.6)	< 0.01
**Medication, n (%)**								
Antiplatelets	225 (27.9)	53 (47.8)	51 (37.0)	58 (26.2)	41 (19.2)	16 (17.6)	6 (18.8)	< 0.01
Calcium channel blockers	377 (46.7)	53 (47.8)	71 (51.5)	104 (47.1)	92 (43.0)	40 (44.0)	17 (53.1)	0.65
ACEI or ARB	319 (39.5)	56 (50.5)	71 (51.5)	93 (42.1)	56 (26.2)	28 (30.8)	15 (46.9)	< 0.01
β-blockers	194 (24.0)	36 (32.4)	42 (30.4)	58 (26.2)	32 (15.0)	19 (20.9)	7 (21.9)	< 0.01
Diuretics	105 (13.0)	24 (21.6)	23 (16.7)	26 (11.8)	13 (6.1)	14 (15.4)	5 (15.6)	< 0.01
Statins	287 (35.6)	51 (46.0)	56 (40.6)	76 (34.4)	57 (26.6)	36 (39.6)	11 (34.4)	0.01
**Medically treated diabetes mellitus**								
Any	176 (21.8)	31 (27.9)	31 (22.5)	43 (19.5)	46 (21.5)	22 (24.2)	3 (9.4)	0.23
Insulin dependent	24 (3.0)	9 (8.1)	6 (4.4)	3 (1.4)	2 (0.9)	2 (2.2)	2 (6.3)	< 0.01

*HDL* indicates high-density lipoprotein, *LDL* low-density lipoprotein, *BUN* blood urea nitrogen, *eGFR* estimated-glomerular filtration rate, *ACEI* angiotensin-converting enzyme inhibitor, *ARB* angiotensin II receptor blocker.

Results are presented as means ± SD for continuous variables and percentages for categorical variables.

We divided the subjects into six groups according to Hct levels. The baseline characteristics of subjects in the six groups are summarized in Table 1. There were significant differences among the six groups according to Hct levels in age, BMI, diastolic blood pressure, total cholesterol, triglycerides, high-density lipoprotein (HDL) cholesterol, low-density lipoprotein (LDL) cholesterol, HbA1c, estimated glomerular filtration rate (eGFR), prevalence of hypertension, prevalence of previous coronary heart disease, prevalence of previous stroke, current smokers, use of antiplatelets, use of an angiotensin-converting enzyme inhibitor or an angiotensin II receptor blocker, use of β-blockers, use of diuretics, use of statins, and use of insulin. There were no significant differences in other parameters among the six groups. Hematologic parameters are summarized in Table 2. There were significant differences among the six groups according to Hct levels in Hb, RBCs, mean corpuscular Hb concentration and platelets. There was no significant difference in other parameters among the six groups.

**Table 2:** Hematologic parameters of the subjects according to hematocrit levels.

Variables	Total (n = 807)	Hematocrit < 37.0% (n = 111)	Hematocrit 37.0–39.9% (n = 138)	Hematocrit 40.0–42.9% (n = 221)	Hematocrit 43.0–45.9% (n = 214)	Hematocrit 46.0–48.9% (n = 91)	Hematocrit 49.0% ≤ (n = 32)	P value
Hemoglobin, g/dL	14.3 ± 1.6	11.7 ± 0.8	13.2 ± 0.5	14.2 ± 0.5	15.3 ± 0.5	16.3 ± 0.5	17.2 ± 0.7	< 0.01
Hematocrit, %	41.8 ± 4.3	34.3 ± 2.3	38.7 ± 0.9	41.4 ± 0.8	44.4 ± 0.8	47.2 ± 0.8	50.4 ± 1.2	
Red blood cell, ×10^6^/μL	4.6 ± 0.5	3.8 ± 0.3	4.2 ± 0.2	4.6 ± 0.3	4.9 ± 0.3	5.2 ± 0.3	5.6 ± 0.2	< 0.01
Mean corpuscular volume, fL	91.1 ± 4.8	91.5 ± 6.0	91.6 ± 4.4	90.6 ± 4.7	91.2 ± 4.7	90.8 ± 4.2	90.8 ± 4.1	0.35
Mean corpuscular hemoglobin, pg	31.2 ± 1.8	31.1 ± 2.2	31.3 ± 1.7	31.0 ± 1.8	31.3 ± 1.8	31.3 ± 1.5	31.0 ± 1.5	0.53
Mean corpuscular hemoglobin concentration, g/dL	34.3 ± 1.1	34.1 ± 1.1	34.1 ± 1.0	34.3 ± 1.0	34.4 ± 0.9	34.5 ± 1.0	34.4 ± 2.2	0.02
Platelets, × 10^3^/μL	205.3 ± 52.8	197.4 ± 53.4	195.5 ± 51.6	207.5 ± 54.1	217.6 ± 48.1	201.7 ± 56.9	190.2 ± 47.2	< 0.01
Mean platelet volume, fL	10.2 ± 0.9	10.2 ± 1.0	10.1 ± 0.8	10.1 ± 0.9	10.2 ± 0.8	10.3 ± 1.0	10.5 ± 0.7	0.14

The baseline characteristics of subjects in the six groups according to Hb and RBC levels are summarized in the Supplemental Tables S1–S4 and are presented in the Supplemental Results section.

### Relationships of Hct, Hb, and RBCs with vascular function

**Figure 1:**
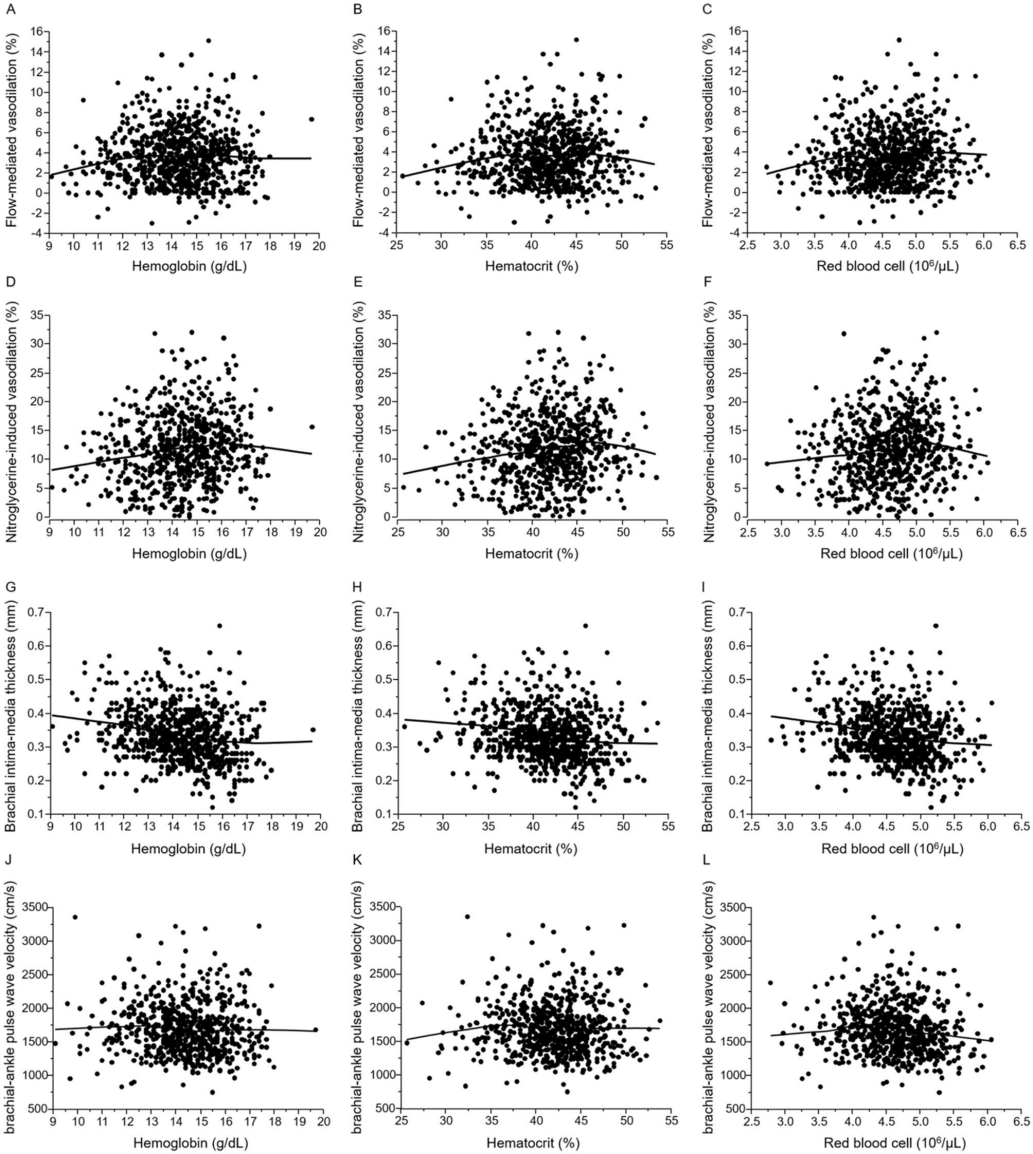
Scatter plots show the relationships of hemoglobin (**A**), hematocrit (**B**), and red blood cell (**C**) with flow-mediated vasodilation, the relationships of hemoglobin (**D**), hematocrit (**E**), and red blood cell (**F**) with nitroglycerine-induced vasodilation, the relationships of hemoglobin (**G**), hematocrit (**H**), and red blood cell (**I**) with brachial intima-media thickness and the relationships of hemoglobin (**J**), hematocrit (**K**), and red blood cell (**L**) with brachial-ankle pulse wave velocity.

Scatter plots between vascular function and hematologic parameters with a Lowess smoothed curve are shown in Fig. 1. Both FMD and NID gradually increased up to Hct levels of about 46–48% and then decreased with further increase in Hct levels. NID was highest in the 46.0–48.9% Hct group among the six groups (10.0 ± 5.3% in the < 37.0% Hct group, 10.8 ± 5.9% in the 37.0–39.9% Hct group, 12.0 ± 6.1% in the 40.0–42.9% Hct group, 12.0 ± 5.7% in the 43.0–45.9% Hct group, 14.2 ± 5.6% in the 46.0–48.9% Hct group and 10.4 ± 4.5% in the ≥ 49.0% Hct group; P < 0.01; Supplemental Figure S1A). There were no significant differences in FMD among the six groups (3.3 ± 2.6% in the < 37.0% Hct group, 3.4 ± 2.6% in the 37.0–39.9% Hct group, 3.6 ± 2.6% in the 40.0–42.9% Hct group, 3.7 ± 2.6% in the 43.0–45.9% Hct group, 3.9 ± 3.1% in the 46.0–48.9% Hct group and 2.9 ± 2.7% in the ≥ 49.0% Hct group; P = 0.39; Supplemental Figure S1B). We used 46.0–48.9% Hct as a reference to define the lower tertile. After adjustment for age, BMI, current smoking and presence of hypertension, dyslipidemia, and diabetes mellitus, adjusted odds ratio of being in the low tertile of NID was significantly higher in the < 42.9% and ≥ 49.0% Hct groups (Table 3). There was no significant difference in low tertile of NID between the 43.0–45.9% Hct group and the 46.0–48.9% Hct group (Table 3).

**Table 3:** Multiple analysis of relationships between low nitroglycerine-induced vasodilation and variables.

Variables	Hematocrit < 37.0%		Hematocrit 37.0–39.9%		Hematocrit 40.0–42.9%		Hematocrit 43.0–45.9%		Hematocrit 46.0–48.9%		Hematocrit 49.0% ≤	
	OR (95% CI)	P value	OR (95% CI)	P value	OR (95% CI)	P value	OR (95% CI)	P value	OR (95% CI)	P value	OR (95% CI)	P value
Unadjusted model	4.1 (2.10–7.87)	< 0.01	2.7 (1.44–5.07)	< 0.01	2.2 (1.24–4.01)	< 0.01	1.9 (1.04–3.38)	0.04	1 (reference)		4.6 (1.91–11.05)	< 0.01
Model 1	2.7 (1.36–5.42)	< 0.01	2.0 (1.04–3.81)	0.04	1.9 (1.03–3.41)	0.04	1.7 (0.92–3.04)	0.09	1 (reference)		4.8 (1.95–11.57)	< 0.01
Model 2	3.2 (1.57–6.46)	< 0.01	2.2 (1.11–4.19)	0.02	2.0 (1.09–3.72)	0.03	1.8 (0.96–3.24)	0.06	1 (reference)		4.3 (1.73–10.49)	< 0.01

Low tertile of nitroglycerine-induced vasodilation indicates less than 10.4%. Model 1: adjusted for age. Model 2: adjusted for age, body mass index, current smoking, hypertension, dyslipidemia and diabetes mellitus.

Clinical characteristics and hematologic parameters of the subjects with Hct of < 48.9% are summarized in Supplemental Tables S5 and S6. Hct was positively correlated with FMD and NID (r = 0.08, P = 0.03 and r = 0.18, P < 0.01, respectively; Supplemental Table S7). Multivariate analysis revealed that Hct was an independent variable of NID (β = 0.11, P < 0.01; Supplemental Table S8). Hct was not an independent variable of FMD (β = − 0.01, P = 0.73; Supplemental Table S9). Hct of 42.0% was the optimal cut-off value for the low tertile of NID (sensitivity, 58.4%; specificity, 55.9%). Characteristics and hematologic parameters of the subjects with Hct of > 46.0% are summarized in Supplemental Tables S10 and S11. Hct was negatively correlated with FMD and NID (r  = − 0.11, P = 0.24 and r =   − 0.25, P < 0.01, respectively; Supplemental Table S12). Multivariate analysis revealed that Hct was an independent variable of NID in multivariate analysis (β = − 0.23, P = 0.01; Supplemental Table S13). Hct was not an independent variable of FMD (β = − 0.07, P = 0.39; Supplemental Table S14). Hct of 49.4% was the optimal cut-off value for the low tertile of NID (sensitivity, 46.0%; specificity, 88.2%).

The relationships of Hb and RBCs with vascular function are presented in the Supplemental Results section, Supplemental Figures S2 and S3 and Supplemental Tables S15–S35.

### Relationships of Hct, Hb, and RBCs with vascular structure

Scatter plots between vascular structure and hematologic parameters with a Lowess smoothed curve are shown in Fig. 1. Brachial IMT significantly decreased in relation to an increase in the levels of Hct categories (0.36 ± 0.08 mm, 0.36 ± 0.07 mm, 0.34 ± 0.07 mm, 0.32 ± 0.08 mm, 0.31 ± 0.08 mm and 0.32 ± 0.08 mm; P < 0.01; Supplemental Figure S4A). There were no significant differences in baPWV among the six groups (1714 ± 410 cm/s, 1729 ± 374 cm/s, 1662 ± 384 cm/s, 1647 ± 385 cm/s, 1702 ± 388 cm/s and 1752 ± 494 cm/s; P = 0.35; Supplemental Figure S4B). We used 46.0–48.9% Hct as a reference to define the lower tertile. After adjustment for age, BMI, current smoking and presence of hypertension, dyslipidemia, and diabetes mellitus, adjusted odds ratio of being in the low tertile of brachial IMT was significantly lower in the < 39.9% Hct groups (Table 4). There were no significant differences in the low tertile of brachial IMT among the 40.0–42.9% Hct group, 43.0–45.9% Hct group, and ≥ 49.0% Hct group and 46.0–48.9% Hct group.

**Table 4:** Multiple analysis of relationships between low brachial intima-media thickness and variables.

Variables	Hematocrit < 37.0%		Hematocrit 37.0–39.9%		Hematocrit 40.0–42.9%		Hematocrit 43.0–45.9%		Hematocrit 46.0–48.9%		Hematocrit 49.0% ≤	
	OR (95% CI)	P value	OR (95% CI)	P value	OR (95% CI)	P value	OR (95% CI)	P value	OR (95% CI)	P value	OR (95% CI)	P value
Unadjusted model	0.2 (0.10–0.39)	< 0.01	0.2 (0.10–0.39)	< 0.01	0.4 (0.25–0.72)	< 0.01	0.7 (0.43–1.21)	0.22	1 (reference)		0.6 (0.26–1.50)	0.29
Model 1	0.4 (0.20–0.83)	0.01	0.3 (0.17–0.65)	< 0.01	0.6 (0.34–1.04)	0.07	0.9 (0.52–1.57)	0.72	1 (reference)		0.6 (0.23–1.49)	0.27
Model 2	0.4 (0.18–0.78)	< 0.01	0.3 (0.18–0.62)	< 0.01	0.6 (0.32–1.01)	0.06	0.9 (0.49–1.52)	0.61	1 (reference)		0.6 (0.24–1.59)	0.32

Low tertile of brachial intima-media thickness indicates less than 0.30 mm. Model 1: adjusted for age. Model 2: adjusted for age, body mass index, current smoking, hypertension, dyslipidemia and diabetes mellitus.

The relationships of Hb and RBCs with vascular structure are presented in the Supplemental Results section, Supplemental Figures S5 and S6 and Supplemental Tables S36–S38.

## Discussion

In the present study, we demonstrated for the first time that Hct, Hb and RBCs were associated with vascular function and vascular structure in men. Adjusted odds ratio of being in the low tertile of NID was significantly higher in the < 42.9% and ≥ 49.0% Hct groups. Adjusted odds ratio of being in the low tertile of NID was significantly higher in the < 13 g/dL Hb group, 14.0–14.9 g/dL Hb group and ≥ 17.0 g/dL Hb group. Adjusted odds ratio of being in the low tertile of NID was significantly higher in the < 4.19 × 10^6^/μL and ≥ 5.40 × 10^6^/μL RBCs groups. In addition, adjusted odds ratio of being in the low tertile of brachial IMT was significantly lower in the < 39.9% Hct groups than in the 46.0–48.9% Hct group. Adjusted odds ratio of being in the low tertile of brachial IMT was significantly lower in the < 14.9 g/dL Hb groups than in the 16.0–16.9 g/dL Hb group. Hct of 42.0–49.4%, Hb of 14.7–16.8 g/dL and RBCs of 4.82–5.24 × 10^6^/μL may be the optimal target levels for maintenance of vascular function and vascular structure.

In the present study, Hct of 42.0–49.4% was best from the aspect of vascular smooth muscle function. Several studies have shown that high Hct levels were associated with an increased risk of cardiovascular disease^1–3^. On the other hand, the relationship between low Hct levels and cardiovascular disease is controversial. Gagnon et al. showed that there were J- or U-shaped relations between Hct and morbidity and mortality from cardiovascular events^1^. After risk factor adjustment, there was a significantly increased risk of cardiovascular disease in the high Hct group but not in the low Hct group in men. Gotoh et al. showed that low Hct levels were associated with hemorrhagic stroke^3^. The effects of Hct, Hb and RBCs on vascular function and vascular structure are unclear. In the present study, we demonstrated that both low and high levels of Hct, Hb and RBCs were associated with vascular smooth muscle dysfunction. Vosseler et al. showed that blood viscosity, which was calculated using account Hct and plasma proteins, was negatively correlated with FMD in patients without coronary artery disease, while there was no significant relationship between blood viscosity and FMD in patients with atheroclerosis^15^. The discrepancy in the result of our study and the results of previous studies regarding the relationship between vascular function and hematocrit is due to the different numbers of subjects and different characteristics of subjects. The number of subjects was larger in the present study than in the previous studies. Our study participants were enrolled from a general population including patients with cardiovascular disease. Interestingly, Giannattasio et al. showed that acute decreases in Hct from 39.9 ± 0.8% to 37.1 ± 0.4% and Hb from 13.3 ± 0.3 to 12.2 ± 0.4 g/dL, after removal of 500 mL of blood and infusion of 500 mL of saline, impaired vascular function in patients with hemochromatosis^16^. In subjects with Hct of < 48.9%, Hct was positively correlated with FMD and NID and Hct was an independent predictor of NID. These findings suggest that subjects with high or low levels of Hct, Hb and RBCs have a high risk of vascular dysfunction and prognostic atherosclerosis.

Some possible mechanisms underlying the association of low Hct with vascular smooth muscle function are postulated. It is possible that oxygen delivery dynamics at the levels of hemoglobin and hematocrit are associated with vascular function. Thorling et al. showed that Hct positively correlated with tissue tension of oxygen even within normal ranges of Hct levels, suggesting that a decrease in Hct leads to a decrease in oxygen supply to tissues^17^. Takemoto et al. showed that hypoxia decreased endothelial NO synthase (eNOS) expression via the activation of Rho-associated kinase^18^. Chronic hypoxia affects endothelial dysfunction via increases in inflammation and oxidative stress^19,20^. Several studies showed that Hct significantly correlated with viscosity^21,22^. Hct is one of the most important factors affecting blood viscosity. In addition, blood viscosity regulates shear stress, which is an inducer of NO production from the endothelium. Martini et al. showed that animals with increased Hct had increased plasma nitrate/nitrite concentrations compared with those in control animals and in eNOS knockout mice through an increase in blood viscosity^22^. These findings suggest that a low level of Hct is harmful for vascular function.

Some possible mechanisms underlying the association of excessively high Hct with vascular smooth muscle dysfunction are postulated. Lewis et al. showed that the patients with excessive erythrocytosis caused by chronic mountain sickness in Andean highlanders had endothelial dysfunction that was partially reversible during oxygen inhalation, suggesting that chronic hypoxia may induce endothelial dysfunction in patients with excessive erythrocytosis^23^. In addition, high blood viscosity caused by high levels of Hct as well as low blood viscosity caused by low levels of Hct induced low tissue tension of oxygen. According to the Hagen-Poiseuille law, blood flow depends on blood viscosity and vessel radius. Total peripheral vascular resistance is specified by blood viscosity and cardiac output. Fowler et al. showed that high viscosity caused low cardiac output^24^. These findings suggest that high levels of Hct may induce tissue tension of oxygen by high peripheral vascular resistance and low cardiac output. These findings also suggest that a high level of Hct may be one of the factors of vascular dysfunction.

It has been shown that RBCs directly affect endothelial function via the eNOS/NO pathway and NOS-like bioactivity and the production of reactive oxygen species^25–27^. Cortese-Krott et al. showed that RBCs contained eNOS and produced NO in healthy subjects as well as in patients with coronary artery disease and that FMD significantly correlated with the expression of eNOS and eNOS activity in RBCs in those subjects^25^. In addition, Zhou et al. showed new mechanisms by which endothelial function was impaired in type 2 diabetes mellitus through activation of RBC arginase 1 and increase in production of reactive oxygen species^27^. These findings suggest that RBC function per se plays an important role in the pathogenesis, maintenance, and development of atherosclerosis through the regulation of vascular function, leading to cardiovascular disease and cardiovascular events. Unfortunately, our study had no information on the function of RBCs, such as the eNOS/NO pathway, NOS like activity and oxidative stress. Assessment of RBC function would enable more specific conclusions concerning the role of RBCs other than the number of RBCs in vascular function to be drawn.

Simply, NID is assessed by brachial artery response to sublingual administration of nitroglycerine. However, we believe that vascular response to exogenous NO reflects vascular smooth muscle function since NO finally acts on vascular smooth muscle cells. Indeed, NID has been widely used as an indicator of vascular smooth muscle function. Several investigators have shown that vascular response to nitric acid including nitroglycerine reflects vascular smooth muscle function in the brachial artery and coronary artery of humans and in the isolated aorta artery of experimental animals^28–30^. It has been shown that NID is impaired in patients with multiple cardiovascular risk factors and that it serves as an independent predictor of cardiovascular events^12,31^. We believe that reduction in vascular smooth muscle response assessed by NID can also be defined vascular smooth muscle dysfunction.

Recently, some trials have shown that patients with type 2 diabetes mellitus who received an inhibitor of sodium-glucose cotransporter 2 in addition to conventional therapy had significantly lower rates of cardiovascular morbidity and mortality than did patients with type 2 diabetes mellitus who received a placebo in addition to conventional therapy^32–34^. The EMPA-REG OUTCOME trial showed that changes in Hct (increase by 5.0 ± 5.3% from baseline of 41.3 ± 5.7%) and Hb (increase by 0.8 ± 1.3 g/dL from baseline of 13.5 ± 1.5 g/dL) within normal ranges might be important mediators of the empagliflozin-induced reduction in incidence of cardiovascular events including cardiovascular mortality^35^. In the present study, Hct was positively correlated with FMD and NID in subjects with Hct < 48.9%, which was an independent variable of NID in multivariate analysis. In addition, Hct level of 42.0–49.4%, Hb level of 14.7–16.8 g/dL and RBC level of 4.82–5.24 × 10^6^/μL may be the optimal target levels for maintenance of vascular function. An increase in the level of Hct up to 49.4% may reduce the incidence of cardiovascular events.

In the present study, adjusted odds ratio of being in the low tertile of brachial IMT was significantly lower in the < 37.0% Hct group and 37.0–39.9% Hct group than in the 46.0–48.9% Hct group and was significantly lower in the < 13.9 g/dL Hb groups and 14.0–14.9 g/dL Hb group than in the 16.0–16.9 g/dL Hb group. Adjusted odds ratio of being in the low tertile of baPWV was significantly lower in the level < 3.80 × 10^6^/μL RBCs group and 4.60–4.99 × 10^6^/μL RBCs group than in the 5.00–5.39 × 10^6^/μL RBCs group. Lee et al. showed that carotid IMT positively correlated with blood viscosity and Hct. In their study, blood viscosity was an independent variable of carotid IMT in multivariate analysis, while Hct was not an independent variable of carotid IMT^5^. Kawamoto et al. showed that Hb levels were not associated with baPWV in men^36^. Unfortunately, the relationships of Hct, Hb and RBCs with vascular structure are also controversial. The roles of Hct, Hb and RBCs in vascular structure need to be confirmed in future in large clinical trials.

In the present study, the groups with high levels of Hct, Hb and RBCs had vascular smooth muscle dysfunction but not abnormal vascular structure. It is well known that alteration of vascular function occurs before changes in vascular structure. Unfortunately, we had no information on the duration of high levels of Hct, Hb and RBCs. Cohort studies have shown that a high Hct level per se was associated with an increased risk of cardiovascular disease^1–3^. NID may be a more sensitive marker than brachial IMT or baPWV of cardiovascular disease in subjects with high levels of Hct and Hb.

Our study has a number of limitations. First, this study is a cross-sectional design. Therefore, we cannot define causal relationships of Hct, Hb and RBCs with vascular dysfunction and abnormal vascular structure. Further studies are needed to confirm the effects of changes in levels of Hct, Hb and RBCs on vascular function and structure in long-term follow-up periods using a prospective study design. Second, we evaluated the relationships of Hct, Hb and RBCs with vascular function and structure only in men. It is well known that menstrual bleeding affects the levels of Hct, Hb and RBCs. We had no information on menstrual cycle when measuring vascular function and structure. Therefore, we excluded women as study subjects. Further studies are needed to confirm the relationships of levels of Hct, Hb and RBCs with vascular function and structure in women including premenopausal women as well as men after adjustment of the menstrual cycle. Third, we defined vascular dysfunction assessed by FMD and that assessed by NID as low tertiles of FMD and NID. The use of criteria for vascular dysfunction is a better way to calculate the odds ratio. However, diagnostic criteria for endothelial dysfunction assessed by FMD and vascular smooth muscle dysfunction assessed by NID have not been established. Therefore, we used low tertiles of FMD and NID as vascular dysfunction for calculation of the odds ratio.

## Conclusion

Low and high levels of Hct, Hb and RBCs were associated with vascular smooth muscle dysfunction, and low Hct levels were associated with abnormal vascular structure. Increases in the levels of Hct, Hb and RBCs within normal ranges may decrease the risk of cardiovascular disease. Hct level of 43.0–48.9%, Hb level of 14.7–16.8 g/dL and RBCs level of 4.82–5.24 × 10^6^/μL may be the optimal target levels for maintenance of vascular function and vascular structure. Therefore, attention should be given to levels of Hct, Hb and RBCs when caring for patients with low or high levels of Hct, Hb and RBCs*.*

## Methods

### Subjects

Between September 2010 and June 2017, a total of 993 men were recruited for measurement of vascular function from subjects who underwent health-screening examinations or who visited the outpatient clinic at Hiroshima University Hospital. One hundred eighty-six of the 993 men, including 59 patients with infection, 50 patients with advanced cancer, 11 patients with bleeding, 35 patients with end-stage renal disease, 16 patients who had received prednisolone treatment, and 15 patients with hematologic disease, were excluded. Finally, 807 men were enrolled in this study. Hypertension was defined as systolic blood pressure of more than 140 mm Hg or diastolic blood pressure of more than 90 mm Hg in a sitting position, on at least three different occasions. DM was defined according to the American Diabetes Association or a previous diagnosis of diabetes^37,38^. Dyslipidemia was defined according to the third report of the National Cholesterol Education Program^39^.

We divided the subjects into six groups according to Hct levels (< 37.0% group, 37.0–39.9% group, 40.0–42.9% group, 43.0–45.9% group, 46.0–48.9% group and ≥ 49.0% group), six groups according to Hb levels (< 13 g/dL group, 13.0–39.9 g/dL group, 14.0–14.9 g/dL group, 15.0–15.9 g/dL group, 16.0–16.9 g/dL group and ≥ 17.0 g/dL group), and six groups according to RBCs levels (< 3.80 × 10^6^/μL group, 3.80–4.19 × 10^6^/μL group, 4.20–4.59 × 10^6^/μL group, 4.60–4.99 × 10^6^/μL group, 5.00–5.39 × 10^6^/μL group and ≥ 5.40 × 10^6^/μL group).

All methods were carried out in accordance with relevant guidelines and regulations. The Ethics Review Board of Hiroshima University approved the study protocol. Written informed consent for participation in the study was obtained from all of the subjects. All methods were performed in accordance with the relevant guidelines and regulations overseen by the Ethical Committee in Hiroshima University.

### Study protocol

We measured vascular function using measurement of FMD and NID and vascular structure using measurement of IMT in the brachial artery and baPWV. Subjects fasted the previous night for at least 12 h and the study began at 8:30 a.m. The subjects were kept in the supine position in a quiet, dark, and air-conditioned room (constant temperature of 22–25°C) throughout the study. A 23-gauge polyethylene catheter was inserted into the left deep antecubital vein to obtain blood samples. After thirty minutes of maintaining the supine position, we measured FMD, NID, brachial IMT and baPWV. The observers were blind to the form of examination^40^. **Clinical trial registration information:** URL for Clinical Trial: https://www.umin.ac.jp Registration Number for Clinical Trial: UMIN000003409.

### Measurements of FMD and NID

Vascular response to reactive hyperemia in the brachial artery was used for assessment of endothelium-dependent FMD. A high-resolution linear artery transducer was coupled to computer-assisted analysis software (UNEXEF18G, UNEX Co, Nagoya, Japan) that used an automated edge detection system for measurement of brachial artery diameter^12^. The response to nitroglycerine was used for assessment of endothelium-independent vasodilation. NID was measured as described previously^12^. Additional details are available in the online-only Data Supplement.

### Measurement of brachial IMT

Before FMD measurement, baseline longitudinal ultrasonographic images of the brachial artery, obtained at the end of diastole from each of 10 cardiac cycles, were automatically stored on a hard disk for off-line assessment of IMT with a linear, phased-array high-frequency (10-MHz) transducer using an UNEXEF18G ultrasound unit (UNEX Co)^13^. Additional details are available in the online-only Data Supplement.

### Measurement of baPWV

Aortic compliance was assessed noninvasively on the basis of Doppler ultrasound measurements of PWV along the descending thoracoabdominal aorta, as previously published and validated^41^. Additional details are available in the online-only Data Supplement.

### Statistical analysis

Results are presented as means ± SD for continuous variables and as percentages for categorical variables. Statistical significance was set at a level of P < 0.05. Categorical variables were compared by means of the χ^2^ test. Continuous variables were compared by ANOVA. Associations between variables were determined by Spearman rank correlation analysis. Associations of FMD, NID, brachial IMT and baPWV with hematologic parameters were examined visually using locally weighted regression smoothing (Lowess) plots. Cut-off values of Hct, Hb and RBCs were evaluated on the basis of receiver-operating characteristic curve analysis using the Youden index. Multivariate regression analysis was performed to identify independent variables associated with low tertiles of FMD (< 2.2%), NID (< 10.4%), brachial IMT (< 0.30 mm) and baPWV (< 1501 cm/s). A reverse U-shaped relation was showed between Hct and NID. We performed formal tests of linearity for the relation between Hct and NID. The R^2^ value of the quadratic model of Hct was better than that of the linear model of Hct (0.023 and 0.019, respectively). Thus, the model with Hct as a quadratic function gives a better fit. Peak Hct of 46.64% was calculated by the delta method. Peak Hct was used to determine the Hct range of 46.0–48.9% at which NID was the highest among the six groups, and the Hct range of 46.0–48.9% was used as the reference group in the multiple logistic regression analysis. Similar reverse U-shaped relations were found between Hb and NID and RBCs and NID. The peaks of Hb and RBCs were used to determine the ranges of 16.0–16.9 g/dL for Hb and 5.00–5.39 × 10^6^/μL for RBCs at which NID values were the highest among the six groups, and the ranges of 16.0–16.9 g/dL for Hb and 5.00–5.39 × 10^6^/μL for RBCs were used as reference groups in the multiple logistic regression analysis. Age, body mass index (BMI), current smoking and presence of hypertension, dyslipidemia, and diabetes mellitus were entered into the multiple logistic regression analysis. The data were processed using JMP pro version 13 (SAS institute. Cary, NC).

## Supplementary information

Supplementary Information.
