# Supplementary material for: Hematocrit, hemoglobin and red blood cells are associated with vascular function and vascular structure in men
Source: Sci Rep. 2020 Jul 10;10:11467. doi: 10.1038/s41598-020-68319-1 (PMC7351756; doi:10.1038/s41598-020-68319-1)
Supplement: Supplementary file 1 — Supplementary Information. [file 41598_2020_68319_MOESM1_ESM.docx]

**Online Supplement**

**Hematocrit, Hemoglobin and Red Blood Cell Are Associated with Vascular Function and Vascular Structure in Men**

Brief title: Hematocrit and vascular function

Shinji Kishimoto, MD, PhD;^1^ Tatsuya Maruhashi, MD, PhD;^2^ Masato Kajikawa, MD, PhD;^3^ Shogo Matsui, MD, PhD;^4^ Haruki Hashimoto, MD;^2^ Yuji Takaeko, MD;^2^ Takahiro Harada, MD;^2^ Takayuki Yamaji, MD;^2^ Yiming Han, MS;^2^ Yasuki Kihara, MD, PhD;^2^ Kazuaki Chayama, MD, PhD;^5^ Chikara Goto, PhD;^6^ Farina Mohamad Yusoff, MD;^1^ Ayumu Nakashima, MD, PhD;^7^ Yukihito Higashi, MD, PhD, FAHA^1,3^

^1^Department of Cardiovascular Regeneration and Medicine, Research Institute for Radiation Biology and Medicine, Hiroshima University, Hiroshima, Japan

^2^Department of Cardiovascular Medicine, Graduate School of Biomedical and Health Sciences, Hiroshima University, Hiroshima, Japan

^3^Division of Regeneration and Medicine, Medical Center for Translational and Clinical Research, Hiroshima University Hospital, Hiroshima, Japan

^4^Department of Cardiovascular Medicine, Hiroshima Prefectural Hospital, Hiroshima, Japan

^5^Department of Gastroenterology and Metabolism, Graduate School of Biomedical and Health Sciences, Hiroshima University Hiroshima, Japan

^6^Dpartment of Rehabilitation, Faculty of General Rehabilitation, Hiroshima International University, Hiroshima, Japan

^7^Department of Stem Cell Biology and Medicine, Graduate School of Biomedical and Health Sciences, Hiroshima University Hiroshima, Japan

Address for correspondence: Yukihito Higashi, MD, PhD, FAHA

Department of Cardiovascular Regeneration and Medicine,

Research Institute for Radiation Biology and Medicine (RIRBM), Hiroshima University

1-2-3 Kasumi, Minami-ku, Hiroshima 734-8551, Japan

Phone: +81-82-257-5831 Fax: +81-82-257-5831

E-mail: [yhigashi@hiroshima-u.ac.jp](mailto:yhigashi@hiroshima-u.ac.jp)

**Methods**

**Measurements of FMD and NID**

Vascular response to reactive hyperemia in the brachial artery was used for assessment of endothelium-dependent FMD. A high-resolution linear artery transducer was coupled to computer-assisted analysis software (UNEXEF18G, UNEX Co, Nagoya, Japan) that used an automated edge detection system for measurement of brachial artery diameter.^1^ A blood pressure cuff was placed around the forearm. The brachial artery was scanned longitudinally 5-10 cm above the elbow. When the clearest B-mode image of the anterior and posterior intimal interfaces between the lumen and vessel wall was obtained, the transducer was held at the same point throughout the scan by a special probe holder (UNEX Co) to ensure consistency of the image. Depth and gain setting were set to optimize the images of the arterial lumen wall interface. When the tracking gate was placed on the intima, the artery diameter was automatically tracked, and the waveform of diameter changes over the cardiac cycle was displayed in real time using the FMD mode of the tracking system. This allowed the ultrasound images to be optimized at the start of the scan and the transducer position to be adjusted immediately for optimal tracking performance throughout the scan. Pulsed Doppler flow was assessed at baseline and during peak hyperemic flow, which was confirmed to occur within 15 seconds after cuff deflation. Blood flow velocity was calculated from the color Doppler data and was displayed as a waveform in real time. The baseline longitudinal image of the artery was acquired for 30 seconds, and then the blood pressure cuff was inflated to 50 mm Hg above systolic pressure for 5 minutes. The longitudinal image of the artery was recorded continuously until 5 minutes after cuff deflation. Pulsed Doppler velocity signals were obtained for 20 seconds at baseline and for 10 seconds immediately after cuff deflation. Changes in brachial artery diameter were immediately expressed as percentage change relative to the vessel diameter before cuff inflation. FMD was automatically calculated as the percentage change in peak vessel diameter from the baseline value. Percentage of FMD [(Peak diameter - Baseline diameter)/Baseline diameter] was used for analysis. Blood flow volume was calculated by multiplying the Doppler flow velocity (corrected for the angle) by heart rate and vessel cross-sectional area (-r2). Reactive hyperemia was calculated as the maximum percentage increase in flow after cuff deflation compared with baseline flow.

The response to nitroglycerine was used for assessment of endothelium-independent vasodilation. NID was measured as described previously.^1^ Briefly, after acquiring baseline rest images for 30 seconds, a sublingual tablet (75 μg nitroglycerine) was given, and images of the artery was recorded continuously until the dilation reached a plateau after administration of nitroglycerine. Subjects who had received nitrate treatment and subjects in whom the sublingually administered nitroglycerine tablet was not dissolved during the measurement were excluded from this study. NID was automatically calculated as a percent change in peak vessel diameter from the baseline value. Percentage of NID [(Peak diameter - Baseline diameter)/Baseline diameter] was used for analysis. Inter- and intra-coefficients of variation for the brachial artery diameter were 1.6% and 1.4%, respectively, in our laboratory.

**Measurement of Brachial IMT**

Before FMD measurement, baseline longitudinal ultrasonographic images of the brachial artery, obtained at the end of diastole from each of 10 cardiac cycles, were automatically stored on a hard disk for off-line assessment of IMT with a linear, phased-array high-frequency (10-MHz) transducer using an UNEXEF18G ultrasound unit (UNEX Co).^2^ Measurement of IMT was automatically performed on A-mode images of the far wall of the brachial artery. The analysis system automatically chose the measurement point where an image of the posterior intimal interface was clearly obtained. If the measurement point was inappropriate, another clear image site could be manually selected for measurement. A total of 21 points over a 3-mm length of IMT in the 10-mm longitudinal image depicted in the analysis display were measured and the mean value per image was automatically calculated. IMT was measured at the same point in each image. The average of mean values obtained from 10 cardiac cycles was defined as IMT of the brachial artery.

When measuring carotid IMT, we had an anatomical landmark, such as the carotid-artery bulb. Unfortunately, it is difficult to measure the same site of the brachial artery attributable to the lack of an anatomical landmark. Measurement of IMT in the brachial artery was performed at the proper site where the clearest B-mode image of the anterior and posterior intimal interfaces between the lumen and vessel wall was obtained at 5 to 10 cm above the elbow. However, there was little influence of intra- and interpatient variability in the measurement location of the brachial artery at 5 to 10 cm above the elbow in the present study, because the interface on the intima media of brachial artery is relatively smooth and intima-media thickening is not localized or plaques are not presented, resulting in diffuse intima-media thickening. The coefficients of variation of intra- and interobserver brachial IMT measurements were 3.1% and 4.0%, respectively.

**Measurement of baPWV**

Aortic compliance was assessed noninvasively on the basis of Doppler ultrasound measurements of PWV along the descending thoracoabdominal aorta, as previously published and validated.^3^ Briefly, baPWV, an index of arterial stiffness, was determined by two pressure sensors placed on the right ankle and left brachial arteries to record each pulse wave simultaneously, and the time lag (t) between the notches of the two waves using a pulse wave velocimeter (Form PWV/ABI, model BP-203RPE, Colin Co.). The distance (D) between the two recording sensors was calculated automatically by inputting the value of individual height. The PWV value was calculated as PWV=D/t. PWV was measured for five consecutive pulses, and averages were used for analysis.

**References**

1. Maruhashi T, Soga J, Fujimura N, Idei N, Mikami S, Iwamoto Y, Kajikawa M, Matsumoto T, Hidaka T, Kihara Y, Chayama K, Noma K, Nakashima A, Goto C, Higashi Y. Nitroglycerine-induced vasodilation for assessment of vascular function: A comparison with flow-mediated vasodilation. *Arteriosclerosis, thrombosis, and vascular biology*. 2013;33:1401-1408.
2. Iwamoto Y, Maruhashi T, Fujii Y, Idei N, Fujimura N, Mikami S, Kajikawa M, Matsumoto T, Kihara Y, Chayama K, Noma K, Nakashima A, Higashi Y. Intima-media thickness of brachial artery, vascular function, and cardiovascular risk factors. *Arteriosclerosis, thrombosis, and vascular biology*. 2012;32:2295-230.
3. Kimoto E, Shoji T, Shinohara K, Inaba M, Okuno Y, Miki T, Koyama H, Emoto M, Nishizawa Y. Preferential stiffening of central over peripheral arteries in type 2 diabetes. *Diabetes*. 2003;52:448-452.

**Results**

**Baseline Clinical Characteristics**

We divided the subjects into six groups according to Hb levels. The baseline characteristics of subjects in the six groups are summarized in Supplemental Table S1. There were significant differences among the six groups according to Hb levels in age, BMI, systolic blood pressure, heart rate, diastolic blood pressure, total cholesterol, triglycerides, HDL cholesterol, LDL cholesterol, hemoglobin A1c, eGFR, prevalence of hypertension, prevalence of previous coronary heart disease, prevalence of previous stroke, current smoking, use of antiplatelets, use of an angiotensin-converting enzyme inhibitor or an angiotensin II receptor blocker, use of β-blockers, use of diuretics, use of statins, and use of insulin. There was no significant difference in other parameters among the six groups according to Hb levels. Hematologic parameters are summarized in Supplemental Table S2. There were significant differences among the six groups according to Hb levels in hemoglobin, Hct, RBC, mean corpuscular Hb, mean corpuscular Hb concentration and platelets. There was no significant difference in other parameters among the six groups.

Next, we divided the subjects into six groups according to RBC levels. The baseline characteristics of subjects in the six groups are summarized in Supplemental Table S3. There were significant differences among the six groups according to RBC levels in age, BMI, diastolic blood pressure, heart rate, total cholesterol, triglycerides, HDL cholesterol, LDL cholesterol, glucose, hemoglobin A1c, eGFR, prevalence of previous coronary heart disease, current smoking, use of antiplatelets, use of an angiotensin-converting enzyme inhibitor or an angiotensin II receptor blocker, use of β-blockers, use of diuretics, and use of insulin. There was no significant difference in other parameters among the six groups according to RBC levels. Hematologic parameters are summarized in Supplemental Table S4. There were significant differences among the six groups according to RBC levels in Hb, Hct, RBC, mean corpuscular volume, mean corpuscular Hb, mean corpuscular Hb concentration and platelets. There was no significant difference in other parameters among the six groups.

**Relationships of Hb and RBCs with Vascular Function**

Scatter plots between vascular function and hematologic parameters with a Lowess smoothed curve are shown in Figure 1. Both FMD and NID gradually increased up to Hb levels of about 16.0-16.9 g/dL and then decreased with further increase in Hb levels. NID was highest in the 16-16.9 g/dL group of the six groups according to Hb levels (9.9±5.2% in the <13 g/dL Hb group, 11.7±6.0% in the 13.0-39.9 g/dL Hb group, 11.9±5.3% in the 14.0-14.9 g/dL Hb group, 12.0±5.3% in the 15.0-15.9 g/dL Hb group, 14.3±5.5% in the 16.0-16.9 g/dL Hb group and 10.8±4.8% in the ≥17.0 g/dL Hb group; P<0.01; Supplemental Figure S2A). There were no significant differences in FMD among the six groups according to Hb levels (3.3±2.4% in the <13 g/dL Hb group, 3.5±2.7% in the 13.0-39.9 g/dL Hb group, 3.7±2.6% in the 14.0-14.9 g/dL Hb group, 3.6±2.8% in the 15.0-15.9 g/dL Hb group, 3.7±2.8% in the 16.0-16.9 g/dL Hb group and 3.2±2.6% in the ≥17.0 g/dL Hb group; P=0.83; Supplemental Figure S2B). We used 16.0-16.9 g/dL Hb as a reference to define the lower tertile. After adjustment for age, BMI, current smoking and presence of hypertension, dyslipidemia, and diabetes mellitus, adjusted odds ratio of being in the low tertile of NID was significantly higher in the <13 g/dL Hb group, in the 14.0-14.9 g/dL Hb group and in the ≥17.0 g/dL group (Supplemental Table S15). There were no significant differences in the low tertile of NID among the 13.0-13.9 g/dL Hb group, 15.0-15.9 g/dL Hb group and 16.0-16.9 g/dL Hb group (Supplemental Table S15).

Clinical characteristics and hematologic parameters of the subjects with Hb of <17 g/dL are summarized in Supplemental Tables S16 and S17. Hb was positively correlated with FMD and NID (r=0.06, P=0.11 and r=0.18, P<0.01, respectively; Supplemental Table S18). Hb was not correlated with FMD (r=0.06, P=0.11; Supplemental Table S18) Multivariate analysis revealed that Hb was an independent variable of NID (β=0.11, P<0.01; supplemental Table S19). Hb of 14.7 g/dL was the optimal cut-off value for the low tertile of NID (sensitivity, 70.4%; specificity, 46.2%). Clinical characteristics and hematologic parameters of the subjects with Hb of >16.0 g/dL are summarized in Supplemental Tables S20 and S21. Hb negatively correlated with FMD and NID (r=-0.03, P=0.75 and r=-0.23, P=0.02, respectively; Supplemental Table S22). Hb was not correlated with FMD (r=0.03, P=0.75; Supplemental Table S22) Multivariate analysis revealed that Hb was an independent variable of NID (β=-0.21, P=0.03; Supplemental Table S23). Hb of 16.8 g/dL was the optimal cut-off value for the low tertile of NID (sensitivity, 64.7%; specificity, 71.4%).

Scatter plots between vascular function and hematologic parameters with a Lowess smoothed curve are shown in Figure 1. Both FMD and NID gradually increased up to RBC levels of about 5.00-5.39 x 10^6^/μL and then decreased with further increase in RBC level. FMD was highest in the 5.00-5.39 x 10^6^/μL RBCs group among the six groups according to RBCs levels (2.6±2.2% in the <3.80 x 10^6^/μL RBCs group, 3.7±2.6% in the 3.80-4.19 x 10^6^/μL RBCs group, 3.3±2.6% in the 4.20-4.59 x 10^6^/μL RBCs group, 3.6±2.6% in the 5.00-5.39 x 10^6^/μL RBCs group, 4.0±2.8% in the 5.00-5.39 x 10^6^/μL RBCs group and 3.8±2.8% in the ≥5.40 x 10^6^/μL RBCs group; P<0.01; Supplemental Figure S3A). We used 5.00-5.39 x 10^6^/μL RBCs as a reference to define the lower tertile. After adjustment for age, BMI, current smoking and presence of hypertension, dyslipidemia, and diabetes mellitus, there was no significant difference in adjusted odds ratio of the low tertile of FMD among the six groups according to RBC levels (Supplemental Table S24). NID was highest in the 5.00-5.39 x 10^6^/μL group among the six groups according to RBCs (10.2±5.0% in the <3.80 x 10^6^/μL RBCs group, 10.5±5.8% in the 3.80-4.19 x 10^6^/μL RBCs group, 11.7±6.0% in the 4.20-4.59 x 10^6^/μL RBCs group, 11.6±5.7% in the 5.00-5.39 x 10^6^/μL RBCs group, 13.7±5.9% in the 5.00-5.39 x 10^6^/μL RBCs group and 11.0±5.1% in the ≥5.40 x 10^6^/μL RBCs group; P<0.01; Supplemental Figure S3B). After adjustment for age, BMI, current smoking and presence of hypertension, dyslipidemia, and diabetes mellitus, adjusted odds ratio of being in the low tertile of NID was significantly higher in the <4.19 x 10^6^/μL and ≥5.40 x 10^6^/μL RBCs groups (Supplemental Table S25). There were no significant differences in the low tertile of NID among the 4.20-4.59 x 10^6^/μL RBCs group, 4.60-4.99 x 10^6^/μL RBCs group and 5.00-5.39 x 10^6^/μL RBCs group (Supplemental Table S25).

Clinical characteristics and hematologic parameters of the subjects with RBCs of <5.40 x 10^6^/μL are summarized in Supplemental Tables S26 and S27. RBCs were positively correlated with FMD and NID (r=0.09, P<0.01 and r=0.16, P<0.01, respectively; Supplemental Table S28). Multivariate analysis revealed that RBC level was an independent variable of NID (β=0.09, P=0.03; Supplemental Table S29). RBC level was not an independent variable of FMD (β=-0.003, P=0.97; Supplemental Table S30). RBC level of 4.82 x 10^6^/μL was the optimal cut-off value for the low tertile of NID (sensitivity, 75.9%; specificity, 38.9%). Clinical characteristics and hematologic parameters of the subjects with RBCs of >5.00 x 10^6^/μL are summarized in Supplemental Tables S31 and S32. RBC level was negatively correlated with FMD and NID (r=-0.05, P=0.48 and r=-0.17, P=0.02, respectively; Table S33). RBC level was not correlated with FMD (r=-0.05, P=0.48; Table S33) Multivariate analysis revealed that RBC level was an independent variable of NID (β=-0.22, P<0.01; Supplemental Table S34). RBC level was not an independent variable of FMD (β=-0.006, P=0.37) (Supplemental Table S35). RBCs value of 5.24 x 10^6^/μL was the optimal cut-off value for the low tertile of NID (sensitivity, 64.9%; specificity, 62.9%).

**Relationships of Hb and RBC with Vascular Structure**

Brachial IMT decreased significantly in relation to an increase in the levels of Hb categories (0.36±0.08 mm, 0.35±0.08 mm, 0.33±0.07 mm, 0.32±0.08 mm, 0.31±0.09 mm and 0.31±0.07 mm; P<0.01; Supplemental Figure S5A). There were no significant differences in baPWV among the six groups (1726±411 cm/s, 1697±327 cm/s, 1661±387 cm/s, 1671±392 cm/s, 1689±383 cm/s and 1632±437 cm/s; P=0.72; Supplemental Figure S5B). After adjustment for age, BMI, current smoking and presence of hypertension, dyslipidemia, and diabetes mellitus, adjusted odds ratio of being in the low tertile of brachial IMT was significantly lower in the <14.9 g/dL Hb groups than in the 16.0-16.9 g/dL Hb group (Supplemental Table S36). There were no significant differences in the low tertile of brachial IMT among the 15.0-15.9 g/dL group, ≥17.0 g/dL group and 16.0-16.9 g/dL group (Supplemental Table S36).

Brachial IMT and baPWV decreased significantly in relation to an increase in the levels of RBC categories (0.36±0.08 mm, 0.35±0.08 mm, 0.33±0.07 mm, 0.32±0.08 mm, 0.31±0.09 mm and 0.31±0.07 mm; P<0.01 and 1627±379 cm/s, 1754±325 cm/s, 1754±399 cm/s, 1676±380 cm/s, 1599±368 cm/s and 1600±443 cm/s; P<0.01; Supplemental Figure S6A and S6B). After adjustment for age, BMI, current smoking and presence of hypertension, dyslipidemia, and diabetes mellitus, adjusted odds ratio of being in the low tertile of baPWV was significantly lower in the <3.80 x 10^6^/μL RBCs group and in the 4.60-4.99 x 10^6^/μL RBCs group than in the 5.00-5.39 x 10^6^/μL RBCs group (Supplemental Table S37). There were no significant differences in the low tertile of baPWV among the 3.80-4.19 x 10^6^/μL RBCs group, 4.20-4.59 x 10^6^/μL RBCs group, ≥5.40 x 10^6^/μL RBCs group and 5.00-5.39 x 10^6^/μL RBCs group (Supplemental Table S37). After adjustment for age, BMI, current smoking and presence of hypertension, dyslipidemia, and diabetes mellitus, there were no significant differences in adjusted odds ratio of the low tertile of brachial IMT among the six groups (Supplemental Table S38).

**Supplemental Tables**

**Table S1**. Clinical Characteristics of the Subjects According to Hemoglobin Levels

| Variables | Total  (n = 807) | Hemoglobin  < 13.0 g/dL  (n = 153) | Hemoglobin  13.0-13.9 g/dL  (n = 166) | Hemoglobin  14.0-14.9 g/dL  (n = 191) | Hemoglobin  15.0-15.9 g/dL  (n = 178) | Hemoglobin  16.0-16.9 g/dL  (n = 85) | Hemoglobin  17.0 g/dL ≤  (n = 34) | P value |
| --- | --- | --- | --- | --- | --- | --- | --- | --- |
| Age, yr | 62±14 | 70±12 | 67±10 | 61±14 | 58±14 | 54±14 | 51±12 | <0.01 |
| Body mass index, kg/m^2^ | 24.7±3.9 | 23.3±3.8 | 24.3±3.0 | 24.4±3.7 | 25.5±4.1 | 25.7±4.1 | 27.6±4.2 | <0.01 |
| Systolic blood pressure, mmHg | 134±19 | 135±21 | 132±18 | 131±19 | 135±18 | 135±21 | 141±18 | 0.01 |
| Diastolic blood pressure, mmHg | 80±12 | 76±11 | 78±11 | 80±12 | 82±12 | 82±13 | 87±11 | <0.01 |
| Heart rate, bpm | 70±13 | 68±13 | 68±12 | 70±12 | 70±13 | 75±14 | 71±12 | <0.01 |
| Total cholesterol, mmol/L | 4.86±0.96 | 4.47±0.83 | 4.63±0.83 | 4.91±0.93 | 5.07±0.96 | 5.30±0.98 | 5.22±1.06 | <0.01 |
| Triglycerides, mmol/L | 1.70±1.20 | 1.37±0.97 | 1.51±0.91 | 1.60±1.05 | 1.87±1.13 | 2.09±1.42 | 2.87±2.36 | <0.01 |
| HDL cholesterol, mmol/L | 1.47±0.44 | 1.55±0.54 | 1.47±0.47 | 1.42±0.39 | 1.42±0.39 | 1.47±0.39 | 1.27±0.31 | <0.01 |
| LDL cholesterol, mmol/L | 2.84±0.85 | 2.48±0.72 | 2.64±0.67 | 2.95±0.93 | 3.03±0.85 | 3.13±0.98 | 3.15±0.70 | <0.01 |
| Glucose, mmol/L | 6.77±5.11 | 6.88±2.66 | 6.77±1.89 | 6.61±2.28 | 6.72±2.39 | 6.49±2.11 | 7.99±5.11 | 0.09 |
| Hemoglobin A1c, % | 5.8±1.0 | 6.0±1.4 | 5.8±0.7 | 5.7±0.7 | 5.8±0.9 | 5.7±1.0 | 6.4±1.9 | 0.01 |
| BUN, mmol/L | 5.71±1.93 | 7.14±2.75 | 6.07±1.93 | 5.36±1.32 | 5.36±1.36 | 5.36±1.36 | 5.36±1.93 | <0.01 |
| Creatinine, mmol/L | 81.3±25.6 | 96.4±45.1 | 79.6±17.7 | 75.1±18.6 | 75.1±18.6 | 76.9±15.0 | 81.3±20.3 | <0.01 |
| eGFR, ml/min/1.73 m^2^ | 71±20 | 63±24 | 69±17 | 72±16 | 77±19 | 76±16 | 75±20 | <0.01 |
| Medical history, n (%) |  |  |  |  |  |  |  |  |
| Hypertension | 627 (77.7) | 126 (82.4) | 141 (84.9) | 136 (71.2) | 133 (74.7) | 62 (72.9) | 29 (85.3) | 0.01 |
| Dyslipidemia | 496 (61.5) | 85 (55.6) | 110 (66.3) | 114 (59.7) | 112 (62.9) | 50 (58.8) | 25 (73.5) | 0.25 |
| Diabetes mellitus | 269 (33.3) | 60 (39.2) | 52 (31.3) | 59 (30.9) | 61 (34.3) | 26 (30.6) | 11 (32.4) | 0.62 |
| Previous coronary heart disease | 171 (21.2) | 51 (33.3) | 55 (33.1) | 28 (14.7) | 19 (10.7) | 10 (11.8) | 8 (23.5) | <0.01 |
| Previous stroke | 70 (8.7) | 28 (18.3) | 15 (9.0) | 9 (4.7) | 9 (5.1) | 6 (7.1) | 3 (8.8) | <0.01 |
| Current smoker, n (%) | 188 (23.3) | 23 (15.0) | 17 (10.2) | 51 (26.7) | 53 (29.8) | 28 (32.9) | 16 (47.1) | <0.01 |
| Medication, n (%) |  |  |  |  |  |  |  |  |
| Antiplatelets | 225 (27.9) | 67 (43.8) | 68 (41.0) | 40 (20.9) | 36 (20.2) | 8 (9.4) | 6 (17.7) | <0.01 |
| Calcium channel blockers | 377 (46.7) | 75 (49.0) | 82 (49.4) | 81 (42.4) | 87 (48.9) | 34 (40.0) | 18 (52.9) | 0.44 |
| ACEI or ARB | 319 (39.5) | 79 (51.6) | 86 (51.8) | 70 (36.7) | 41 (23.0) | 30 (35.3) | 13 (38.2) | <0.01 |
| β-blockers | 194 (24.0) | 52 (34.0) | 57 (34.3) | 37 (19.4) | 24 (13.5) | 16 (18.8) | 8 (23.5) | <0.01 |
| Diuretics | 105 (13.0) | 35 (22.9) | 26 (15.7) | 18 (9.4) | 14 (7.9) | 10 (11.8) | 2 (5.9) | <0.01 |
| Statins | 287 (35.6) | 68 (44.4) | 72 (43.4) | 63 (33.0) | 49 (27.5) | 24 (28.2) | 11 (32.4) | 0.01 |
| Medically treated diabetes mellitus |  |  |  |  |  |  |  |  |
| Any | 176 (21.8) | 40 (26.1) | 36 (21.7) | 38 (19.9) | 41 (23.0) | 18 (21.2) | 3 (8.8) | 0.49 |
| Insulin dependent | 24 (3.0) | 12 (7.8) | 5 (3.0) | 0 (0.0) | 5 (2.8) | 0 (0.0) | 2 (5.9) | <0.01 |

HDL indicates high-density lipoprotein; LDL, low-density lipoprotein; BUN, blood urea nitrogen; eGFR, estimated-glomerular filtration rate; ACEI, angiotensin-converting enzyme inhibitor; ARB, angiotensin II receptor blocker.

Results are presented as means±SD for continuous variables and percentages for categorical variables.

**Table S2**. Hematologic Parameters of the Subjects According to Hemoglobin Levels

| Variables | Total  (n = 807) | Hemoglobin  < 13.0 g/dL  (n = 153) | Hemoglobin  13.0-13.9 g/dL  (n = 166) | Hemoglobin  14.0-14.9 g/dL  (n = 191) | Hemoglobin  15.0-15.9 g/dL  (n = 178) | Hemoglobin  16.0-16.9 g/dL  (n = 85) | Hemoglobin  17.0 g/dL ≤  (n = 34) | P value |
| --- | --- | --- | --- | --- | --- | --- | --- | --- |
| Hemoglobin, g/dL | 14.3±1.6 | 11.9±0.8 | 13.5±0.3 | 14.5±0.3 | 15.4±0.3 | 16.4±0.3 | 17.4±0.5 | <0.01 |
| Hematocrit, % | 41.8±4.3 | 35.4±2.7 | 39.7±1.3 | 42.2±1.3 | 44.8±1.5 | 46.8±1.5 | 49.7±1.4 | <0.01 |
| Red blood cell, x 10^6^/μL | 4.6±0.5 | 3.9±0.4 | 4.3±0.3 | 4.7±0.3 | 4.9±0.3 | 5.1±0.2 | 5.6±0.3 | <0.01 |
| Mean corpuscular volume, fL | 91.1±4.8 | 91.4±5.6 | 91.7±4.4 | 90.7±4.6 | 90.9±4.9 | 91.1±3.9 | 89.3±3.8 | 0.08 |
| Mean corpuscular hemoglobin, pg | 31.2±1.8 | 30.8±2.2 | 31.1±1.9 | 31.1±1.6 | 31.3±1.7 | 31.9±1.5 | 31.4±1.6 | <0.01 |
| Mean corpuscular hemoglobin concentration, g/dL | 34.3±1.1 | 33.7±1.1 | 34.0±0.9 | 34.3±0.8 | 34.5±1.0 | 35.0±1.0 | 35.3±1.7 | <0.01 |
| Platelets, x 10^3^/μL | 205.3±52.8 | 194.1±51.3 | 198.5±50.0 | 215.1±57.8 | 209.7±52.8 | 206.8±46.5 | 209.9±48.8 | <0.01 |
| Mean platelet volume, fL | 10.2±0.9 | 10.3±1.0 | 10.1±0.9 | 10.1±0.8 | 10.2±0.8 | 10.4±0.8 | 10.4±0.8 | 0.10 |

**Table S3**. Clinical Characteristics of the Subjects According to Red Blood Cell Levels

| Variables | Total  (n = 807) | Red blood cell  < 3.80 x 10^6^/μL  (n = 55) | Red blood cell  3.80-4.19  x 10^6^/μL  (n = 124) | Red blood cell  4.20-4.59  x 10^6^/μL  (n = 203) | Red blood cell  4.60-4.99  x 10^6^/μL  (n = 238) | Red blood cell  5.00-5.39  x 10^6^/μL  (n = 140) | Red blood cell  5.40  x 10^6^/μL ≤  (n = 47) | P value |
| --- | --- | --- | --- | --- | --- | --- | --- | --- |
| Age, yr | 62±14 | 74±10 | 69±10 | 65±11 | 61±13 | 52±15 | 52±14 | <0.01 |
| Body mass index, kg/m^2^ | 24.7±3.9 | 22.7±4.0 | 23.7±2.9 | 23.9±3.5 | 24.9±3.6 | 26.4±4.3 | 26.7±4.6 | <0.01 |
| Systolic blood pressure, mmHg | 134±19 | 130±21 | 135±19 | 133±19 | 133±19 | 136±20 | 135±19 | 0.33 |
| Diastolic blood pressure, mmHg | 80±12 | 71±10 | 77±11 | 80±11 | 83±12 | 83±13 | 81±12 | <0.01 |
| Heart rate, bpm | 70±13 | 69±15 | 67±12 | 69±12 | 71±13 | 71±12 | 71±13 | 0.047 |
| Total cholesterol, mmol/L | 4.86±0.96 | 4.32±0.80 | 4.65±0.88 | 4.68±0.83 | 4.97±0.93 | 5.28±1.01 | 5.07±1.14 | <0.01 |
| Triglycerides, mmol/L | 1.70±1.20 | 1.38±1.26 | 1.41±0.81 | 1.55±1.04 | 1.69±0.97 | 2.03±1.41 | 2.60±2.07 | <0.01 |
| HDL cholesterol, mmol/L | 1.47±0.44 | 1.58±0.52 | 1.58±0.52 | 1.53±0.47 | 1.42±0.36 | 1.34±0.34 | 1.34±0.34 | <0.01 |
| LDL cholesterol, mmol/L | 2.84±0.85 | 2.28±0.65 | 2.61±0.78 | 2.66±0.75 | 2.97±0.83 | 3.26±0.91 | 2.97±0.96 | <0.01 |
| Glucose, mmol/L | 6.77±2.66 | 6.88±2.50 | 6.88±2.50 | 6.61±1.67 | 6.94±2.83 | 6.22±1.50 | 7.60±4.50 | 0.02 |
| Hemoglobin A1c, % | 5.8±1.0 | 6.0±0.8 | 5.9±1.5 | 5.7±0.7 | 5.8±1.0 | 5.7±0.8 | 6.2±1.6 | 0.04 |
| BUN, mmol/L | 5.71±1.93 | 7.85±3.46 | 6.78±2.14 | 5.71±1.64 | 5.36±1.46 | 5.36±1.39 | 5.36±1.64 | <0.01 |
| Creatinine, mmol/L | 81.3±20.3 | 102.5±25.6 | 86.6±37.1 | 79.6±22.1 | 76.9±18.6 | 76.9±15.0 | 80.4±17.7 | <0.01 |
| eGFR, ml/min/1.73 m^2^ | 71±20 | 55±21 | 67±22 | 70±18 | 74±18 | 77±19 | 74±18 | <0.01 |
| Medical history, n (%) |  |  |  |  |  |  |  |  |
| Hypertension | 627 (77.7) | 39 (70.9) | 107 (86.3) | 161 (79.3) | 177 (74.4) | 106 (75.7) | 37 (78.7) | 0.11 |
| Dyslipidemia | 496 (61.5) | 33 (60.0) | 74 (59.7) | 117 (57.6) | 148 (62.2) | 92 (65.7) | 32 (68.1) | 0.63 |
| Diabetes mellitus | 269 (33.3) | 24 (43.7) | 44 (35.5) | 66 (32.5) | 83 (34.9) | 33 (23.6) | 19 (40.4) | 0.07 |
| Previous coronary heart disease | 171 (21.2) | 21 (38.2) | 39 (31.5) | 48 (23.7) | 33 (13.9) | 19 (13.6) | 11 (23.4) | <0.01 |
| Previous stroke | 70 (8.7) | 8 (14.6) | 18 (14.5) | 16 (7.9) | 16 (6.7) | 8 (5.7) | 4 (8.5) | 0.06 |
| Current smoker, n (%) | 188 (23.3) | 10 (18.2) | 22 (17.7) | 36 (17.7) | 66 (27.7) | 40 (28.6) | 14 (29.8) | 0.03 |
| Medication, n (%) |  |  |  |  |  |  |  |  |
| Antiplatelets | 225 (27.9) | 28 (50.9) | 51 (41.1) | 65 (32.0) | 47 (19.8) | 23 (16.4) | 11 (23.4) | <0.01 |
| Calcium channel blockers | 377 (46.7) | 20 (36.4) | 64 (51.6) | 99 (48.8) | 113 (47.5) | 61 (43.6) | 20 (42.6) | 0.43 |
| ACEI or ARB | 319 (39.5) | 34 (61.8) | 62 (50.0) | 89 (43.8) | 75 (31.5) | 42 (30.0) | 17 (36.2) | <0.01 |
| β-blockers | 194 (24.0) | 23 (41.8) | 38 (30.7) | 65 (32.0) | 33 (13.9) | 25 (17.9) | 10 (21.3) | <0.01 |
| Diuretics | 105 (13.0) | 16 (29.1) | 24 (19.4) | 23 (11.3) | 22 (9.2) | 13 (9.3) | 7 (14.9) | <0.01 |
| Statins | 287 (35.6) | 25 (45.5) | 54 (43.6) | 71 (35.0) | 82 (34.5) | 38 (27.1) | 17 (36.2) | 0.07 |
| Medically treated diabetes mellitus |  |  |  |  |  |  |  |  |
| Any | 176 (21.8) | 17 (30.9) | 27 (21.8) | 41 (20.2) | 59 (24.8) | 23 (16.2) | 10 (21.3) | 0.23 |
| Insulin dependent | 24 (3.0) | 5 (9.1) | 7 (5.7) | 3 (1.5) | 4 (1.7) | 1 (0.7) | 4 (8.5) | <0.01 |

HDL indicates high-density lipoprotein; LDL, low-density lipoprotein; BUN, blood urea nitrogen; eGFR, estimated-glomerular filtration rate; ACEI, angiotensin-converting enzyme inhibitor; ARB, angiotensin II receptor blocker.

Results are presented as means±SD for continuous variables and percentages for categorical variables.

**Table S4**. Hematologic Parameters of the Subjects According to Red Blood Cell Levels

| Variables | Total  (n = 807) | Red blood cell  < 3.80  x 10^6^/μL  (n = 55) | Red blood cell  3.80-4.19  x 10^6^/μL  (n = 124) | Red blood cell  4.20-4.59  x 10^6^/μL  (n = 203) | Red blood cell  4.60-4.99  x 10^6^/μL  (n = 238) | Red blood cell  5.00-5.39  x 10^6^/μL  (n = 140) | Red blood cell  5.40  x 10^6^/μL ≤  (n = 47) | P value |
| --- | --- | --- | --- | --- | --- | --- | --- | --- |
| Hemoglobin, g/dL | 14.3±1.6 | 11.2±0.8 | 12.8±0.7 | 13.8±0.8 | 14.9±0.8 | 15.8±0.8 | 16.8±0.9 | <0.01 |
| Hematocrit, % | 41.8±4.3 | 33.0±2.5 | 37.6±1.9 | 40.6±2.1 | 43.3±1.9 | 45.8±2.0 | 48.7±2.2 | <0.01 |
| Red blood cell, x 10^6^/μL | 4.6±0.5 | 3.5±0.2 | 4.0±0.1 | 4.4±0.1 | 4.8±0.1 | 5.2±0.1 | 5.6±0.2 | <0.01 |
| Mean corpuscular volume, fL | 91.1±4.8 | 94.3±5.9 | 93.9±4.4 | 92.0±4.4 | 90.3±4.0 | 88.8±4.1 | 86.7±3.8 | <0.01 |
| Mean corpuscular hemoglobin, pg | 31.2±1.8 | 32.1±2.0 | 31.9±1.7 | 31.4±1.8 | 31.0±1.8 | 30.6±1.4 | 29.9±1.6 | <0.01 |
| Mean corpuscular hemoglobin concentration, g/dL | 34.3±1.1 | 34.1±1.0 | 34.0±0.9 | 34.1±1.0 | 34.4±1.0 | 34.4±1.1 | 34.7±1.7 | <0.01 |
| Platelets, x 10^3^/μL | 205.3±52.8 | 192.0±54.0 | 188.9±48.1 | 203.9±50.0 | 215.6±52.9 | 209.4±54.7 | 207.0±56.6 | <0.01 |
| Mean platelet volume, fL | 10.2±0.9 | 10.2±1.1 | 10.1±0.8 | 10.2±0.9 | 10.1±0.8 | 10.4±0.9 | 10.3±0.8 | 0.04 |

**Table S5**. Clinical Characteristics of the Subjects in Men with Hematocrit of <48.9%

| Variables | Men  (n = 775) |
| --- | --- |
| Age, yr | 63±14 |
| Body mass index, kg/m^2^ | 24.6±3.8 |
| Systolic blood pressure, mmHg | 133±19 |
| Diastolic blood pressure, mmHg | 79±12 |
| Heart rate, bpm | 70±13 |
| Total cholesterol, mmol/L | 4.86±0.96 |
| Triglycerides, mmol/L | 1.67±1.14 |
| HDL cholesterol, mmol/L | 1.47±0.44 |
| LDL cholesterol, mmol/L | 2.84±0.85 |
| Glucose, mmol/L | 6.72±2.33 |
| Hemoglobin A1c, % | 5.8±1.0 |
| BUN, mmol/L | 5.71±1.93 |
| Creatinine, mmol/L | 81.3±25.6 |
| eGFR, ml/min/1.73 m^2^ | 71±20 |
| Medical history, n (%) |  |
| Hypertension | 598 (77.2) |
| Dyslipidemia | 473 (61.0) |
| Diabetes mellitus | 258 (33.3) |
| Previous coronary heart disease | 161 (20.8) |
| Previous stroke | 67 (8.6) |
| Current smoker, n (%) | 175 (22.7) |
| Medication, n (%) |  |
| Antiplatelets | 219 (28.3) |
| Calcium channel blockers | 360 (46.5) |
| ACEI or ARB | 304 (39.2) |
| β-blockers | 187 (24.1) |
| Diuretics | 100 (12.9) |
| Statins | 276 (35.6) |
| Medically treated diabetes mellitus |  |
| Any | 173 (22.3) |
| Insulin dependent | 22 (2.8) |
| Physiological findings |  |
| FMD, % | 3.6±2.6 |
| Nitroglycerine-induced vasodilation, % | 11.8±5.9 |
| Brachial IMT, mm | 0.34±0.08 |
| baPWV, cm/s | 1681±377 |

HDL indicates high-density lipoprotein; LDL, low-density lipoprotein; BUN, blood urea nitrogen; eGFR, estimated-glomerular filtration rate; ACEI, angiotensin-converting enzyme inhibitor; ARB, angiotensin II receptor blocker; FMD, flow-mediated vasodilation; IMT intima-media thickness; baPWV, brachial-ankle pulse wave velocity.

Results are presented as means±SD for continuous variables and percentages for categorical variables.

**Table S6**. Hematologic Parameters of the Subjects in Men with Hematocrit of <48.9%

| Variables | Men  (n = 775) |
| --- | --- |
| Hemoglobin, g/dL | 14.2±1.5 |
| Hematocrit, % | 41.4±4.0 |
| Red blood cell, x 10^6^/μL | 4.6±0.5 |
| Mean corpuscular volume, fL | 91.1±4.8 |
| Mean corpuscular hemoglobin, pg | 31.2±1.8 |
| Mean corpuscular hemoglobin concentration, g/dL | 34.3±1.0 |
| Platelets, x 10^3^/μL | 206.0±53.0 |
| Mean platelet volume, fL | 10.2±0.9 |

**Table S7**. Univariate Analysis of Relationships among FMD, NID and Variables in Subject with Hematocrit of <48.9%

| Variables | FMD, % | NID, % |
| --- | --- | --- |
| Age, yr | -0.25† | -0.26† |
| Body mass index, kg/m^2^ | -0.03 | -0.02 |
| Systolic blood pressure, mmHg | -0.14 | -0.16† |
| Diastolic blood pressure, mmHg | -0.07 | -0.003 |
| Heart rate, bpm | -0.001 | -0.01 |
| Total cholesterol, mmol/L | -0.03 | 0.13† |
| Triglycerides, mmol/L | -0.08* | 0.07 |
| HDL cholesterol, mmol/L | -0.05 | 0.01 |
| LDL cholesterol, mmol/L | 0.02 | 0.09* |
| Glucose, mmol/L | -0.15† | -0.03 |
| Hemoglobin A1c, % | -0.14† | -0.08 |
| BUN, mmol/L | -0.07 | -0.12† |
| Creatinine, mmol/L | -0.05 | -0.11† |
| eGFR, ml/min/1.73 m^2^ | 0.12† | 0.15† |
| Hemoglobin, g/dL | 0.06 | 0.18† |
| Hematocrit, % | 0.08* | 0.18† |
| Red blood cell, x 10^6^/μL | 0.12† | 0.16† |
| Mean corpuscular volume, fL | -0.09* | 0.01 |
| Mean corpuscular hemoglobin, pg | -0.10† | 0.03 |
| Mean corpuscular hemoglobin concentration, g/dL | -0.04 | 0.05 |
| Platelets, x 10^3^/μL | 0.05 | 0.07 |
| Mean platelet volume, fL | -0.02 | 0.01 |
| FMD, % | - | 0.39† |
| NID, % | -0.39† | - |

*P<0.05, †P<0.01

FMD indicates flow-mediated vasodilation; NID, nitroglycerine-induced vasodilation; HDL, high-density lipoprotein; LDL, low-density lipoprotein; BUN, blood urea nitrogen; eGFR, estimated-glomerular filtration rate.

**Table S8**. Multiple Linear Regression Analysis of Relationships between Nitroglycerine-induced Vasodilation and Variables in Subject with Hematocrit of <48.9%

| Variables | Nitroglycerine-induced vasodilation | |
| --- | --- | --- |
|  | β | P value |
| Age, yr | -0.21 | <0.01 |
| Body mass index, kg/m^2^ | -0.06 | 0.12 |
| Hypertension | -0.15 | <0.01 |
| Dyslipidemia | 0.03 | 0.47 |
| Diabetes mellitus | -0.01 | 0.90 |
| Current smoker | 0.04 | 0.26 |
| Hematocrit, % | 0.11 | <0.01 |

The adjusted r^2^ was 0.11.

**Table S9**. Multiple Linear Regression Analysis of Relationships between Flow-mediated Vasodilation and Variables in Subject with Hematocrit of <48.9%

| Variables | Flow-mediated vasodilation | |
| --- | --- | --- |
|  | β | P value |
| Age, yr | -0.22 | <0.01 |
| Body mass index, kg/m^2^ | -0.04 | 0.36 |
| Hypertension | -0.13 | <0.01 |
| Dyslipidemia | 0.03 | 0.38 |
| Diabetes mellitus | -0.08 | 0.03 |
| Current smoker | 0.03 | 0.36 |
| Hematocrit, % | -0.01 | 0.73 |

The adjusted r^2^ was 0.09.

**Table S10**. Clinical Characteristics of the Subjects in Men with Hematocrit of >46.0%

| Variables | Men  (n = 123) |
| --- | --- |
| Age, yr | 55±13 |
| Body mass index, kg/m^2^ | 26.2±3.8 |
| Systolic blood pressure, mmHg | 137±20 |
| Diastolic blood pressure, mmHg | 83±12 |
| Heart rate, bpm | 72±13 |
| Total cholesterol, mmol/L | 5.09±0.98 |
| Triglycerides, mmol/L | 2.22±1.77 |
| HDL cholesterol, mmol/L | 1.37±0.36 |
| LDL cholesterol, mmol/L | 3.03±0.83 |
| Glucose, mmol/L | 6.94±3.33 |
| Hemoglobin A1c, % | 6.0±1.3 |
| BUN, mmol/L | 5.36±1.57 |
| Creatinine, mmol/L | 78.7±16.8 |
| eGFR, ml/min/1.73 m^2^ | 75±18 |
| Medical history, n (%) |  |
| Hypertension | 96 (78.0) |
| Dyslipidemia | 81 (65.9) |
| Diabetes mellitus | 41 (33.3) |
| Previous coronary heart disease | 24 (19.5) |
| Previous stroke | 9 (7.3) |
| Current smoker, n (%) | 47 (38.2) |
| Medication, n (%) |  |
| Antiplatelets | 22 (17.9) |
| Calcium channel blockers | 57 (46.3) |
| ACEI or ARB | 43 (35.0) |
| β-blockers | 26 (21.1) |
| Diuretics | 19 (15.4) |
| Statins | 47 (38.2) |
| Medically treated diabetes mellitus |  |
| Any | 25 (20.3) |
| Insulin dependent | 4 (3.3) |
| Physiological findings |  |
| FMD, % | 3.6±3.0 |
| Nitroglycerine-induced vasodilation, % | 13.1±5.6 |
| Brachial IMT, mm | 0.31±0.08 |
| baPWV, cm/s | 1715±419 |

HDL indicates high-density lipoprotein; LDL, low-density lipoprotein; BUN, blood urea nitrogen; eGFR, estimated-glomerular filtration rate; ACEI, angiotensin-converting enzyme inhibitor; ARB, angiotensin II receptor blocker; FMD, flow-mediated vasodilation; IMT intima-media thickness; baPWV, brachial-ankle pulse wave velocity.

Results are presented as means±SD for continuous variables and percentages for categorical variables.

**Table S11**. Hematologic Parameters of the Subjects in Men with Hematocrit of >46.0%

| Variables | Men  (n = 123) |
| --- | --- |
| Hemoglobin, g/dL | 16.5±0.7 |
| Hematocrit, % | 48.0±1.7 |
| Red blood cell, x 10^6^/μL | 5.3±0.3 |
| Mean corpuscular volume, fL | 90.8±4.2 |
| Mean corpuscular hemoglobin, pg | 31.2±1.5 |
| Mean corpuscular hemoglobin concentration, g/dL | 34.5±1.4 |
| Platelets, x 10^3^/μL | 198.7±54.6 |
| Mean platelet volume, fL | 10.4±0.9 |

**Table S12**. Univariate Analysis of Relationships among FMD, NID and Variables in Subject with Hematocrit of >46.0%

| Variables | FMD, % | NID, % |
| --- | --- | --- |
| Age, yr | -0.23* | -0.21* |
| Body mass index, kg/m^2^ | -0.18* | -0.15 |
| Systolic blood pressure, mmHg | -0.22* | -0.35† |
| Diastolic blood pressure, mmHg | -0.26† | -0.19* |
| Heart rate, bpm | -0.10 | 0.003 |
| Total cholesterol, mmol/L | -0.29† | -0.14 |
| Triglycerides, mmol/L | -0.16 | -0.11 |
| HDL cholesterol, mmol/L | -0.16 | 0.03 |
| LDL cholesterol, mmol/L | -0.15 | -0.12 |
| Glucose, mmol/L | -0.17 | 0.01 |
| Hemoglobin A1c, % | -0.16 | -0.12 |
| BUN, mmol/L | -0.04 | -0.09 |
| Creatinine, mmol/L | -0.02 | -0.01 |
| eGFR, ml/min/1.73 m^2^ | 0.12 | 0.11 |
| Hemoglobin, g/dL | -0.07 | -0.16 |
| Hematocrit, % | -0.11 | -0.25† |
| Red blood cell, x 10^6^/μL | 0.06 | -0.21† |
| Mean corpuscular volume, fL | -0.14 | 0.07 |
| Mean corpuscular hemoglobin, pg | -0.13 | 0.09 |
| Mean corpuscular hemoglobin concentration, g/dL | -0.07 | -0.01 |
| Platelets, x 10^3^/μL | -0.04 | 0.12 |
| Mean platelet volume, fL | 0.04 | -0.0002 |
| FMD, % | - | 0.35† |
| NID, % | -0.35† | - |

*P<0.05, †P<0.01

FMD indicates flow-mediated vasodilation; NID, nitroglycerine-induced vasodilation; HDL, high-density lipoprotein; LDL, low-density lipoprotein; BUN, blood urea nitrogen; eGFR, estimated-glomerular filtration rate.

**Table S13**. Multiple Linear Regression Analysis of Relationships between Nitroglycerine-induced Vasodilation and Variables in Subject with Hematocrit of >46.0%

| Variables | Nitroglycerine-induced vasodilation | |
| --- | --- | --- |
|  | β | P value |
| Age, yr | -0.32 | <0.01 |
| Body mass index, kg/m^2^ | -0.16 | 0.12 |
| Hypertension | -0.16 | 0.10 |
| Dyslipidemia | 0.03 | 0.74 |
| Diabetes mellitus | 0.23 | 0.02 |
| Current smoker | -0.06 | 0.50 |
| Hematocrit, % | -0.23 | 0.01 |

The adjusted r^2^ was 0.20.

**Table S14**. Multiple Linear Regression Analysis of Relationships between Flow-mediated Vasodilation and Variables in Subject with Hematocrit of >46.0%

| Variables | Flow-mediated vasodilation | |
| --- | --- | --- |
|  | β | P value |
| Age, yr | -0.25 | <0.01 |
| Body mass index, kg/m^2^ | -0.13 | 0.14 |
| Hypertension | -0.35 | <0.01 |
| Dyslipidemia | -0.05 | 0.56 |
| Diabetes mellitus | 0.18 | 0.06 |
| Current smoker | -0.01 | 0.89 |
| Hematocrit, % | -0.07 | 0.39 |

The adjusted r^2^ was 0.24.

**Table S15**. Multiple Analysis of Relationships between Low Nitroglycerine-induced Vasodilation and Variables

| Variables | Hemoglobin  < 13.0 g/dL | | Hemoglobin  13.0-13.9 g/dL | | Hemoglobin  14.0-14.9 g/dL | | Hemoglobin  15.0-15.9 g/dL | | Hemoglobin  16.0-16.9 g/dL | | Hemoglobin  17.0 g/dL ≤ | |
| --- | --- | --- | --- | --- | --- | --- | --- | --- | --- | --- | --- | --- |
|  | OR  (95% CI) | P value | OR  (95% CI) | P value | OR  (95% CI) | P value | OR  (95% CI) | P value | OR  (95% CI) | P value | OR  (95% CI) | P value |
| Unadjusted model | 3.8  (2.01-7.11) | <0.01 | 2.4  (1.28-4.47) | <0.01 | 2.5  (1.38-4.67) | <0.01 | 1.8  (0.97-3.) | 0.06 | 1 (reference) | | 3.4  (1.42-8.09) | <0.01 |
| Model 1 | 2.5  (1.31-4.89) | <0.01 | 1.7  (0.91-3.29) | 0.10 | 2.2  (1.17-4.03) | 0.01 | 1.6  (0.85-2.96) | 0.15 | 1 (reference) | | 3.8  (1.57-9.19) | <0.01 |
| Model 2 | 2.8  (1.43-5.52) | <0.01 | 1.8  (0.95-3.57) | 0.07 | 2.4  (1.28-4.52) | <0.01 | 1.6  (0.84-3.01) | 0.15 | 1 (reference) | | 3.4  (1.38-8.36) | <0.01 |

Low tertile of nitroglycerine-induced vasodilation indicates less than 10.4%. Model 1: adjusted for age. Model 2: adjusted for age, body mass index, current smoking, hypertension, dyslipidemia and diabetes mellitus.

**Table S16**. Clinical Characteristics of the Subjects in Men with Hemoglobin of <17 g/dL

| Variables | Men  (n = 773) |
| --- | --- |
| Age, yr | 63±14 |
| Body mass index, kg/m^2^ | 24.6±3.8 |
| Systolic blood pressure, mmHg | 133±19 |
| Diastolic blood pressure, mmHg | 79±12 |
| Heart rate, bpm | 70±13 |
| Total cholesterol, mmol/L | 4.86±0.96 |
| Triglycerides, mmol/L | 1.66±1.10 |
| HDL cholesterol, mmol/L | 1.47±0.44 |
| LDL cholesterol, mmol/L | 2.84±0.88 |
| Glucose, mmol/L | 6.72±2.28 |
| Hemoglobin A1c, % | 5.8±1.0 |
| BUN, mmol/L | 5.71±1.93 |
| Creatinine, mmol/L | 81.3±25.6 |
| eGFR, ml/min/1.73 m^2^ | 71±20 |
| Medical history, n (%) |  |
| Hypertension | 597 (77.3) |
| Dyslipidemia | 471 (61.0) |
| Diabetes mellitus | 258 (33.4) |
| Previous coronary heart disease | 163 (21.1) |
| Previous stroke | 67 (8.7) |
| Current smoker, n (%) | 171 (22.1) |
| Medication, n (%) |  |
| Antiplatelets | 219 (28.4) |
| Calcium channel blockers | 358 (46.4) |
| ACEI or ARB | 306 (39.6) |
| β-blockers | 186 (24.1) |
| Diuretics | 103 (13.3) |
| Statins | 276 (35.7) |
| Medically treated diabetes mellitus |  |
| Any | 173 (22.4) |
| Insulin dependent | 22 (2.9) |
| Physiological findings |  |
| FMD, % | 3.6±2.6 |
| Nitroglycerine-induced vasodilation, % | 11.8±5.9 |
| Brachial IMT, mm | 0.34±0.08 |
| baPWV, cm/s | 1686±380 |

HDL indicates high-density lipoprotein; LDL, low-density lipoprotein; BUN, blood urea nitrogen; eGFR, estimated-glomerular filtration rate; ACEI, angiotensin-converting enzyme inhibitor; ARB, angiotensin II receptor blocker; FMD, flow-mediated vasodilation; IMT intima-media thickness; baPWV, brachial-ankle pulse wave velocity.

Results are presented as means±SD for continuous variables and percentages for categorical variables.

**Table S17**. Hematologic Parameters of the Subjects in Men with Hemoglobin of <17 g/dL

| Variables | Men  (n = 773) |
| --- | --- |
| Hemoglobin, g/dL | 14.2±1.5 |
| Hematocrit, % | 41.4±4.1 |
| Red blood cell, x 10^6^/μL | 4.6±0.5 |
| Mean corpuscular volume, fL | 91.1±4.8 |
| Mean corpuscular hemoglobin, pg | 31.2±1.8 |
| Mean corpuscular hemoglobin concentration, g/dL | 34.2±1.0 |
| Platelets, x 10^3^/μL | 205.2±53.0 |
| Mean platelet volume, fL | 10.2±0.9 |

**Table S18**. Univariate Analysis of Relationships among FMD, NID and Variables in Subject with Hemoglobin of <17 g/dL

| Variables | FMD, % | NID, % |
| --- | --- | --- |
| Age, yr | -0.25† | -0.25† |
| Body mass index, kg/m^2^ | -0.03 | -0.03 |
| Systolic blood pressure, mmHg | -0.14† | -0.17† |
| Diastolic blood pressure, mmHg | -0.06 | -0.003 |
| Heart rate, bpm | -0.001 | 0.003 |
| Total cholesterol, mmol/L | -0.02 | 0.13† |
| Triglycerides, mmol/L | -0.08* | 0.07 |
| HDL cholesterol, mmol/L | -0.05 | 0.01 |
| LDL cholesterol, mmol/L | 0.03 | 0.08* |
| Glucose, mmol/L | -0.16† | -0.04 |
| Hemoglobin A1c, % | -0.15† | -0.09 |
| BUN, mmol/L | -0.07 | -0.12† |
| Creatinine, mmol/L | -0.05 | -0.12† |
| eGFR, ml/min/1.73 m^2^ | 0.12† | 0.15† |
| Hemoglobin, g/dL | 0.06 | 0.18† |
| Hematocrit, % | 0.07 | 0.17† |
| Red blood cell, x 10^6^/μL | 0.11† | 0.15† |
| Mean corpuscular volume, fL | -0.10† | 0.01 |
| Mean corpuscular hemoglobin, pg | -0.10† | 0.03 |
| Mean corpuscular hemoglobin concentration, g/dL | -0.03 | 0.05 |
| Platelets, x 10^3^/μL | 0.06 | 0.08* |
| Mean platelet volume, fL | -0.02 | 0.01 |
| FMD, % | - | 0.39† |
| NID, % | 0.39† | - |

*P<0.05, †P<0.01

FMD indicates flow-mediated vasodilation; NID, nitroglycerine-induced vasodilation; HDL, high-density lipoprotein; LDL, low-density lipoprotein; BUN, blood urea nitrogen; eGFR, estimated-glomerular filtration rate.

**Table S19**. Multiple Linear Regression Analysis of Relationships between Nitroglycerine-induced Vasodilation and Variables in Subject with Hemoglobin of <17 g/dL

| Variables | Nitroglycerine-induced vasodilation | |
| --- | --- | --- |
|  | β | P value |
| Age, yr | -0.20 | <0.01 |
| Body mass index, kg/m^2^ | -0.07 | 0.10 |
| Hypertension | -0.15 | <0.01 |
| Dyslipidemia | 0.02 | 0.54 |
| Diabetes mellitus | -0.01 | 0.90 |
| Current smoker | 0.04 | 0.27 |
| Hemoglobin, g/dL | 0.11 | <0.01 |

The adjusted r^2^ was 0.11.

**Table S20**. Clinical Characteristics of the Subjects in Men with Hemoglobin of ≥16 g/dL

| Variables | Men  (n = 119) |
| --- | --- |
| Age, yr | 53±13 |
| Body mass index, kg/m^2^ | 26.3±4.2 |
| Systolic blood pressure, mmHg | 137±21 |
| Diastolic blood pressure, mmHg | 84±13 |
| Heart rate, bpm | 74±13 |
| Total cholesterol, mmol/L | 5.28±1.01 |
| Triglycerides, mmol/L | 2.31±1.75 |
| HDL cholesterol, mmol/L | 1.42±0.39 |
| LDL cholesterol, mmol/L | 3.13±0.91 |
| Glucose, mmol/L | 6.94±3.33 |
| Hemoglobin A1c, % | 5.9±1.4 |
| BUN, mmol/L | 5.36±1.61 |
| Creatinine, mmol/L | 77.8±16.8 |
| eGFR, ml/min/1.73 m^2^ | 76±17 |
| Medical history, n (%) |  |
| Hypertension | 91 (76.5) |
| Dyslipidemia | 75 (63.0) |
| Diabetes mellitus | 37 (31.1) |
| Previous coronary heart disease | 18 (15.1) |
| Previous stroke | 9 (7.6) |
| Current smoker, n (%) | 44 (37.0) |
| Medication, n (%) |  |
| Antiplatelets | 14 (11.8) |
| Calcium channel blockers | 52 (43.7) |
| ACEI or ARB | 43 (36.1) |
| β-blockers | 24 (20.2) |
| Diuretics | 12 (10.1) |
| Statins | 35 (29.4) |
| Medically treated diabetes mellitus |  |
| Any | 21 (17.6) |
| Insulin dependent | 2 (1.7) |
| Physiological findings |  |
| FMD, % | 3.6±2.8 |
| Nitroglycerine-induced vasodilation, % | 13.3±5.5 |
| Brachial IMT, mm | 0.31±0.08 |
| baPWV, cm/s | 1673±398 |

HDL indicates high-density lipoprotein; LDL, low-density lipoprotein; BUN, blood urea nitrogen; eGFR, estimated-glomerular filtration rate; ACEI, angiotensin-converting enzyme inhibitor; ARB, angiotensin II receptor blocker; FMD, flow-mediated vasodilation; IMT intima-media thickness; baPWV, brachial-ankle pulse wave velocity.

Results are presented as means±SD for continuous variables and percentages for categorical variables.

**Table S21**. Hematologic Parameters of the Subjects in Men with Hemoglobin of ≥16 g/dL1

| Variables | Men  (n = 120) |
| --- | --- |
| Hemoglobin, g/dL | 16.7±0.6 |
| Hematocrit, % | 47.6±2.0 |
| Red blood cell, x 10^6^/μL | 5.3±0.3 |
| Mean corpuscular volume, fL | 90.6±3.9 |
| Mean corpuscular hemoglobin, pg | 31.7±1.5 |
| Mean corpuscular hemoglobin concentration, g/dL | 35.1±1.2 |
| Platelets, x 10^3^/μL | 207.7±47.0 |
| Mean platelet volume, fL | 10.4±0.8 |

**Table S22**. Univariate Analysis of Relationships among FMD, NID and Variables in Subject with Hemoglobin of ≥16 g/dL

| Variables | FMD, % | NID, % |
| --- | --- | --- |
| Age, yr | -0.25† | -0.20* |
| Body mass index, kg/m^2^ | -0.24† | -0.16 |
| Systolic blood pressure, mmHg | -0.21* | -0.36† |
| Diastolic blood pressure, mmHg | -0.29† | -0.18 |
| Heart rate, bpm | -0.14 | -0.05 |
| Total cholesterol, mmol/L | -0.13 | -0.02 |
| Triglycerides, mmol/L | -0.14 | -0.14 |
| HDL cholesterol, mmol/L | -0.02 | 0.01 |
| LDL cholesterol, mmol/L | -0.05 | 0.04 |
| Glucose, mmol/L | -0.17 | -0.01 |
| Hemoglobin A1c, % | -0.16 | -0.08 |
| BUN, mmol/L | 0.01 | -0.01 |
| Creatinine, mmol/L | 0.04 | 0.02 |
| eGFR, ml/min/1.73 m^2^ | 0.05 | 0.07 |
| Hemoglobin, g/dL | -0.03 | -0.23* |
| Hematocrit, % | -0.09 | -0.21* |
| Red blood cell, x 10^6^/μL | -0.01 | -0.21* |
| Mean corpuscular volume, fL | -0.06 | 0.10 |
| Mean corpuscular hemoglobin, pg | -0.004 | 0.07 |
| Mean corpuscular hemoglobin concentration, g/dL | -0.03 | -0.04 |
| Platelets, x 10^3^/μL | -0.07 | -0.01 |
| Mean platelet volume, fL | 0.11 | -0.05 |
| FMD, % | - | 0.32† |
| NID, % | 0.32† | - |

*P<0.05, †P<0.01

FMD indicates flow-mediated vasodilation; NID, nitroglycerine-induced vasodilation; HDL, high-density lipoprotein; LDL, low-density lipoprotein; BUN, blood urea nitrogen; eGFR, estimated-glomerular filtration rate.

**Table S23**. Multiple Linear Regression Analysis of Relationships between Nitroglycerine-induced Vasodilation and Variables in Subject with Hemoglobin of ≥16 g/dL

| Variables | Nitroglycerine-induced vasodilation | |
| --- | --- | --- |
|  | β | P value |
| Age, yr | -0.26 | <0.01 |
| Body mass index, kg/m^2^ | -0.13 | 0.18 |
| Hypertension | -0.16 | 0.09 |
| Dyslipidemia | -0.05 | 0.61 |
| Diabetes mellitus | 0.27 | 0.01 |
| Current smoker | -0.08 | 0.40 |
| Hemoglobin, g/dL | -0.21 | 0.03 |

The adjusted r^2^ was 0.18.

**Table S24**. Multiple Analysis of Relationships between Low Flow-mediated Vasodilation and Variables

| Variables | Red blood cell  < 3.80 x 10^6^/μL | | Red blood cell  3.80-4.19 x 10^6^/μL | | Red blood cell  4.20-4.59 x 10^6^/μL | | Red blood cell  4.60-4.99 x 10^6^/μL | | Red blood cell  5.00-5.39 x 10^6^/μL | | Red blood cell  5.40 x 10^6^/μL ≤ | |
| --- | --- | --- | --- | --- | --- | --- | --- | --- | --- | --- | --- | --- |
|  | OR  (95% CI) | P value | OR  (95% CI) | P value | OR  (95% CI) | P value | OR  (95% CI) | P value | OR  (95% CI) | P value | OR  (95% CI) | P value |
| Unadjusted model | 2.8  (1.46-5.31) | <0.01 | 1.3  (0.78-2.25) | 0.29 | 2.0  (1.24-3.14) | <0.01 | 1.3  (0.83-2.09) | 0.24 | 1 (reference) | | 1.4  (0.68-2.82) | 0.37 |
| Model 1 | 1.4  (0.67-2.77) | 0.39 | 0.8  (0.42-1.34) | 0.34 | 1.3  (0.79-2.14) | 0.30 | 1.0  (0.60-1.57) | 0.89 | 1 (reference) | | 1.4  (0.67-2.88) | 0.37 |
| Model 2 | 1.4  (0.65-2.84) | 0.41 | 0.7  (0.41-1.35) | 0.33 | 1.3  (0.78-2.19) | 0.30 | 1.0  (0.58-1.58) | 0.87 | 1 (reference) | | 1.3  (0.60-2.63) | 0.55 |

Low tertile of flow-mediated vasodilation indicates less than 2.2%. Model 1: adjusted for age. Model 2: adjusted for age, body mass index, current smoking, hypertension, dyslipidemia and diabetes mellitus.

**Table S25**. Multiple Analysis of Relationships between Low Nitroglycerine-induced Vasodilation and Variables

| Variables | Red blood cell  < 3.80 x 10^6^/μL | | Red blood cell  3.80-4.19 x 10^6^/μL | | Red blood cell  4.20-4.59 x 10^6^/μL | | Red blood cell  4.60-4.99 x 10^6^/μL | | Red blood cell  5.00-5.39 x 10^6^/μL | | Red blood cell  5.40 x 10^6^/μL ≤ | |
| --- | --- | --- | --- | --- | --- | --- | --- | --- | --- | --- | --- | --- |
|  | OR  (95% CI) | P value | OR  (95% CI) | P value | OR  (95% CI) | P value | OR  (95% CI) | P value | OR  (95% CI) | P value | OR  (95% CI) | P value |
| Unadjusted model | 3.0  (1.47-6.11) | <0.01 | 2.6  (1.49-4.40) | <0.01 | 1.9  (1.14-3.03) | 0.01 | 1.6  (1.03-2.64) | 0.04 | 1 (reference) | | 2.5  (1.22-5.07) | 0.01 |
| Model 1 | 1.7  (0.78-3.59) | 0.19 | 1.6  (0.89-2.87) | 0.11 | 1.3  (0.78-2.20) | 0.31 | 1.3  (0.78-2.09) | 0.34 | 1 (reference) | | 2.6  (1.25-5.36) | 0.01 |
| Model 2 | 2.3  (1.01-5.00) | 0.04 | 2.0  (1.07-3.62) | 0.03 | 1.6  (0.93-2.74) | 0.09 | 1.5  (0.91-2.53) | 0.11 | 1 (reference) | | 2.8  (1.33-5.91) | <0.01 |

Low tertile of nitroglycerine-induced vasodilation indicates less than 10.4%. Model 1: adjusted for age. Model 2: adjusted for age, body mass index, current smoking, hypertension, dyslipidemia and diabetes mellitus.

**Table S26**. Clinical Characteristics of the Subjects in Men with Red Blood Cell of <5.40 x 10^6^/μL

| Variables | Men  (n = 760) |
| --- | --- |
| Age, yr | 63±14 |
| Body mass index, kg/m^2^ | 24.6±3.8 |
| Systolic blood pressure, mmHg | 134±19 |
| Diastolic blood pressure, mmHg | 79±12 |
| Heart rate, bpm | 70±13 |
| Total cholesterol, mmol/L | 4.86±0.93 |
| Triglycerides, mmol/L | 1.65±1.11 |
| HDL cholesterol, mmol/L | 1.47±0.44 |
| LDL cholesterol, mmol/L | 2.84±0.85 |
| Glucose, mmol/L | 6.72±2.33 |
| Hemoglobin A1c, % | 5.8±1.0 |
| BUN, mmol/L | 5.71±1.96 |
| Creatinine, mmol/L | 81.3±25.6 |
| eGFR, ml/min/1.73 m^2^ | 71±20 |
| Medical history, n (%) |  |
| Hypertension | 590 (77.6) |
| Dyslipidemia | 464 (61.1) |
| Diabetes mellitus | 250 (32.9) |
| Previous coronary heart disease | 160 (21.1) |
| Previous stroke | 66 (8.7) |
| Current smoker, n (%) | 174 (22.9) |
| Medication, n (%) |  |
| Antiplatelets | 214 (28.2) |
| Calcium channel blockers | 357 (47.0) |
| ACEI or ARB | 302 (39.7) |
| β-blockers | 184 (24.2) |
| Diuretics | 98 (12.9) |
| Statins | 270 (35.6) |
| Medically treated diabetes mellitus |  |
| Any | 166 (21.8) |
| Insulin dependent | 20 (2.6) |
| Physiological findings |  |
| FMD, % | 3.5±2.6 |
| Nitroglycerine-induced vasodilation, % | 11.7±5.9 |
| Brachial IMT, mm | 0.34±0.08 |
| baPWV, cm/s | 1689±378 |

HDL indicates high-density lipoprotein; LDL, low-density lipoprotein; BUN, blood urea nitrogen; eGFR, estimated-glomerular filtration rate; ACEI, angiotensin-converting enzyme inhibitor; ARB, angiotensin II receptor blocker; FMD, flow-mediated vasodilation; IMT intima-media thickness; baPWV, brachial-ankle pulse wave velocity.

Results are presented as means±SD for continuous variables and percentages for categorical variables.

**Table S27**. Hematologic Parameters of the Subjects in Men with Red Blood Cell of <5.40 x 10^6^/μL

| Variables | Men  (n = 762) |
| --- | --- |
| Hemoglobin, g/dL | 14.2±1.5 |
| Hematocrit, % | 41.4±4.1 |
| Red blood cell, x 10^6^/μL | 4.5±0.5 |
| Mean corpuscular volume, fL | 91.3±4.7 |
| Mean corpuscular hemoglobin, pg | 31.3±1.8 |
| Mean corpuscular hemoglobin concentration, g/dL | 34.2±1.0 |
| Platelets, x 10^3^/μL | 205.2±52.6 |
| Mean platelet volume, fL | 10.2±0.9 |

**Table S28**. Univariate Analysis of Relationships among FMD, NID and Variables in Subject with Red Blood Cell of <5.40 x 10^6^/μL

| Variables | FMD, % | NID, % |
| --- | --- | --- |
| Age, yr | -0.24† | -0.26† |
| Body mass index, kg/m^2^ | -0.04 | -0.04 |
| Systolic blood pressure, mmHg | -0.14† | -0.18† |
| Diastolic blood pressure, mmHg | -0.08* | -0.02 |
| Heart rate, bpm | 0.0002 | -0.01 |
| Total cholesterol, mmol/L | -0.03 | 0.13† |
| Triglycerides, mmol/L | -0.09* | 0.08 |
| HDL cholesterol, mmol/L | -0.04 | 0.02 |
| LDL cholesterol, mmol/L | 0.02 | 0.08 |
| Glucose, mmol/L | -0.16† | -0.04 |
| Hemoglobin A1c, % | -0.16† | -0.09 |
| BUN, mmol/L | -0.07 | -0.12† |
| Creatinine, mmol/L | -0.05 | -0.12† |
| eGFR, ml/min/1.73 m^2^ | 0.12† | 0.15† |
| Hemoglobin, g/dL | 0.04 | 0.18† |
| Hematocrit, % | 0.05 | 0.17† |
| Red blood cell, x 10^6^/μL | 0.09† | 0.16† |
| Mean corpuscular volume, fL | -0.08* | 0.01 |
| Mean corpuscular hemoglobin, pg | -0.09* | 0.03 |
| Mean corpuscular hemoglobin concentration, g/dL | -0.04 | 0.04 |
| Platelets, x 10^3^/μL | 0.05 | 0.08* |
| Mean platelet volume, fL | -0.02 | -0.002 |
| FMD, % | - | 0.39† |
| NID, % | 0.39† | - |

*P<0.05, †P<0.01

FMD indicates flow-mediated vasodilation; NID, nitroglycerine-induced vasodilation; HDL, high-density lipoprotein; LDL, low-density lipoprotein; BUN, blood urea nitrogen; eGFR, estimated-glomerular filtration rate.

**Table S29**. Multiple Linear Regression Analysis of Relationships between Nitroglycerine-induced Vasodilation and Variables in Subject with Red Blood Cell of <5.40 x 10^6^/μL

| Variables | Nitroglycerine-induced vasodilation | |
| --- | --- | --- |
|  | β | P value |
| Age, yr | -0.21 | <0.01 |
| Body mass index, kg/m^2^ | -0.08 | 0.04 |
| Hypertension | -0.16 | <0.01 |
| Dyslipidemia | 0.03 | 0.38 |
| Diabetes mellitus | -0.003 | 0.94 |
| Current smoker | 0.04 | 0.28 |
| Red blood cell, x 10^6^/μL | 0.09 | 0.03 |

The adjusted r^2^ was 0.11.

**Table S30**. Multiple Linear Regression Analysis of Relationships between Flow-mediated Vasodilation and Variables in Subject with Red Blood Cell of <5.40 x 10^6^/μL

| Variables | Flow-mediated vasodilation | |
| --- | --- | --- |
|  | β | P value |
| Age, yr | -0.22 | <0.01 |
| Body mass index, kg/m^2^ | -0.05 | 0.17 |
| Hypertension | -0.14 | <0.01 |
| Dyslipidemia | 0.05 | 0.22 |
| Diabetes mellitus | -0.09 | 0.02 |
| Current smoker | 0.02 | 0.50 |
| Red blood cell, x 10^6^/μL | -0.003 | 0.97 |

The adjusted r^2^ was 0.09.

**Table S31**. Clinical Characteristics of the Subjects in Men with Red Blood Cell of ≥5.00 x 10^6^/μL

| Variables | Men  (n = 187) |
| --- | --- |
| Age, yr | 52±15 |
| Body mass index, kg/m^2^ | 26.5±4.4 |
| Systolic blood pressure, mmHg | 136±20 |
| Diastolic blood pressure, mmHg | 83±13 |
| Heart rate, bpm | 71±12 |
| Total cholesterol, mmol/L | 5.22±1.03 |
| Triglycerides, mmol/L | 2.18±1.61 |
| HDL cholesterol, mmol/L | 1.34±0.34 |
| LDL cholesterol, mmol/L | 3.18±0.93 |
| Glucose, mmol/L | 6.55±2.66 |
| Hemoglobin A1c, % | 5.8±1.2 |
| BUN, mmol/L | 5.36±1.46 |
| Creatinine, mmol/L | 77.8±15.9 |
| eGFR, ml/min/1.73 m^2^ | 77±19 |
| Medical history, n (%) |  |
| Hypertension | 143 (76.5) |
| Dyslipidemia | 124 (66.3) |
| Diabetes mellitus | 52 (27.8) |
| Previous coronary heart disease | 30 (16.0) |
| Previous stroke | 12 (6.4) |
| Current smoker, n (%) | 54 (28.9) |
| Medication, n (%) |  |
| Antiplatelets | 34 (18.2) |
| Calcium channel blockers | 81 (43.3) |
| ACEI or ARB | 59 (31.6) |
| β-blockers | 35 (18.8) |
| Diuretics | 20 (10.7) |
| Statins | 55 (29.4) |
| Medically treated diabetes mellitus |  |
| Any | 32 (17.1) |
| Insulin dependent | 5 (2.7) |
| Physiological findings |  |
| FMD, % | 4.0±2.8 |
| Nitroglycerine-induced vasodilation, % | 13.0±5.8 |
| Brachial IMT, mm | 0.31±0.08 |
| baPWV, cm/s | 1599±387 |

HDL indicates high-density lipoprotein; LDL, low-density lipoprotein; BUN, blood urea nitrogen; eGFR, estimated-glomerular filtration rate; ACEI, angiotensin-converting enzyme inhibitor; ARB, angiotensin II receptor blocker; FMD, flow-mediated vasodilation; IMT intima-media thickness; baPWV, brachial-ankle pulse wave velocity.

Results are presented as means±SD for continuous variables and percentages for categorical variables.

**Table S32**. Hematologic Parameters of the Subjects in Men with Red Blood Cell of ≥5.00 x 10^6^/μL

| Variables | Men  (n = 189) |
| --- | --- |
| Hemoglobin, g/dL | 16.0±0.9 |
| Hematocrit, % | 46.6±2.4 |
| Red blood cell, x 10^6^/μL | 5.3±0.2 |
| Mean corpuscular volume, fL | 88.2±4.1 |
| Mean corpuscular hemoglobin, pg | 30.4±1.5 |
| Mean corpuscular hemoglobin concentration, g/dL | 34.5±1.3 |
| Platelets, x 10^3^/μL | 208.8±55.0 |
| Mean platelet volume, fL | 10.4±0.9 |

**Table S33**. Univariate Analysis of Relationships among FMD, NID and Variables in Subject with Red Blood Cell of ≥5.00 x 10^6^/μL

| Variables | FMD, % | NID, % |
| --- | --- | --- |
| Age, yr | -0.24† | -0.28† |
| Body mass index, kg/m^2^ | -0.21† | -0.20† |
| Systolic blood pressure, mmHg | -0.26† | -0.34† |
| Diastolic blood pressure, mmHg | -0.26† | -0.16* |
| Heart rate, bpm | -0.002 | 0.03 |
| Total cholesterol, mmol/L | -0.17* | -0.03 |
| Triglycerides, mmol/L | -0.12 | -0.08 |
| HDL cholesterol, mmol/L | -0.10 | -0.01 |
| LDL cholesterol, mmol/L | -0.08 | -0.01 |
| Glucose, mmol/L | -0.23† | -0.10 |
| Hemoglobin A1c, % | -0.17* | -0.11 |
| BUN, mmol/L | -0.04 | -0.11 |
| Creatinine, mmol/L | -0.06 | -0.12 |
| eGFR, ml/min/1.73 m^2^ | 0.16* | 0.22† |
| Hemoglobin, g/dL | -0.16* | -0.10 |
| Hematocrit, % | -0.18* | -0.16* |
| Red blood cell, x 10^6^/μL | -0.05 | -0.17* |
| Mean corpuscular volume, fL | -0.14 | -0.01 |
| Mean corpuscular hemoglobin, pg | -0.14 | 0.01 |
| Mean corpuscular hemoglobin concentration, g/dL | -0.07 | 0.02 |
| Platelets, x 10^3^/μL | -0.04 | 0.03 |
| Mean platelet volume, fL | 0.08 | 0.04 |
| FMD, % | - | 0.39† |
| NID, % | 0.39† | - |

*P<0.05, †P<0.01

FMD indicates flow-mediated vasodilation; NID, nitroglycerine-induced vasodilation; HDL, high-density lipoprotein; LDL, low-density lipoprotein; BUN, blood urea nitrogen; eGFR, estimated-glomerular filtration rate.

**Table S34**. Multiple Linear Regression Analysis of Relationships between Nitroglycerine-induced Vasodilation and Variables in Subject with Red Blood Cell of ≥5.00 x 10^6^/μL

| Variables | Nitroglycerine-induced vasodilation | |
| --- | --- | --- |
|  | β | P value |
| Age, yr | -0.31 | <0.01 |
| Body mass index, kg/m^2^ | -0.18 | 0.02 |
| Hypertension | -0.25 | <0.01 |
| Dyslipidemia | 0.03 | 0.69 |
| Diabetes mellitus | 0.17 | 0.046 |
| Current smoker | -0.07 | 0.34 |
| Red blood cell, x 10^6^/μL | -0.22 | <0.01 |

The adjusted r^2^ was 0.23.

**Table S35**. Multiple Linear Regression Analysis of Relationships between Flow-mediated Vasodilation and Variables in Subject with Red Blood Cell of ≥5.00 x 10^6^/μL

| Variables | Flow-mediated vasodilation | |
| --- | --- | --- |
|  | β | P value |
| Age, yr | -0.21 | 0.01 |
| Body mass index, kg/m^2^ | -0.15 | 0.06 |
| Hypertension | -0.24 | <0.01 |
| Dyslipidemia | -0.06 | 0.40 |
| Diabetes mellitus | 0.07 | 0.41 |
| Current smoker | -0.07 | 0.30 |
| Red blood cell, x 10^6^/μL | -0.06 | 0.37 |

The adjusted r^2^ was 0.17.

**Table S36**. Multiple Analysis of Relationships between Low Brachial Intima-media Thickness and Variables

| Variables | Hemoglobin  < 13.0 g/dL | | Hemoglobin  13.0-13.9 g/dL | | Hemoglobin  14.0-14.9 g/dL | | Hemoglobin  15.0-15.9 g/dL | | Hemoglobin  16.0-16.9 g/dL | | Hemoglobin  17.0 g/dL ≤ | |
| --- | --- | --- | --- | --- | --- | --- | --- | --- | --- | --- | --- | --- |
|  | OR  (95% CI) | P value | OR  (95% CI) | P value | OR  (95% CI) | P value | OR  (95% CI) | P value | OR  (95% CI) | P value | OR  (95% CI) | P value |
| Unadjusted model | 0.2  (0.09-0.32) | <0.01 | 0.2  (0.11-0.36) | <0.01 | 0.4  (0.25-0.74) | <0.01 | 0.6  (0.33-0.99) | 0.047 | 1 (reference) | | 0.7  (0.28-1.59) | 0.36 |
| Model 1 | 0.3  (0.17-0.63) | <0.01 | 0.3  (0.19-0.66) | <0.01 | 0.6  (0.31-0.99) | 0.048 | 0.7  (0.38-1.21) | 0.19 | 1 (reference) | | 0.5  (0.20-1.25) | 0.14 |
| Model 2 | 0.3  (0.15-0.60) | <0.01 | 0.3  (0.18-0.64) | <0.01 | 0.5  (0.29-0.94) | 0.03 | 0.7  (0.37-1.19) | 0.17 | 1 (reference) | | 0.5  (0.20-1.31) | 0.16 |

Low tertile of brachial intima-media thickness indicates less than 0.30 mm. Model 1: adjusted for age. Model 2: adjusted for age, body mass index, current smoking, hypertension, dyslipidemia and diabetes mellitus.

**Table S37**. Multiple Analysis of Relationships between Low brachial-ankle Pulse Wave Velocity and Variables

| Variables | Red blood cell  < 3.80 x 10^6^/μL | | Red blood cell  3.80-4.19 x 10^6^/μL | | Red blood cell  4.20-4.59 x 10^6^/μL | | Red blood cell  4.60-4.99 x 10^6^/μL | | Red blood cell  5.00-5.39 x 10^6^/μL | | | Red blood cell  5.40 x 10^6^/μL ≤ | |
| --- | --- | --- | --- | --- | --- | --- | --- | --- | --- | --- | --- | --- | --- |
|  | OR  (95% CI) | P value | OR  (95% CI) | P value | OR  (95% CI) | P value | OR  (95% CI) | P value | OR  (95% CI) | P value | OR  (95% CI) | | P value |
| Unadjusted model | 1.2  (0.56-2.44) | 0.68 | 3.0  (1.60-5.42) | <0.01 | 2.1  (1.25-3.38) | <0.01 | 1.3  (0.81-2.04) | 0.28 | 1 (reference) | | 0.69  (0.34-1.41) | | 0.69 |
| Model 1 | 0.2  (0.06-0.40) | <0.01 | 0.8  (0.38-1.61) | 0.51 | 0.7  (0.37-1.21) | 0.18 | 0.5  (0.31-0.94) | 0.03 | 1 (reference) | | 0.6  (0.29-1.45) | | 0.29 |
| Model 2 | 0.2  (0.06-0.41) | <0.01 | 0.7  (0.32-1.42) | 0.30 | 0.7  (0.35-1.23) | 0.19 | 0.5  (0.29-0.84) | 0.02 | 1 (reference) | | 0.6  (0.24-1.32) | | 0.19 |

Low tertile of brachial-ankle Pulse Wave Velocity indicates less than 1501 cm/s. Model 1: adjusted for age. Model 2: adjusted for age, body mass index, current smoking, hypertension, dyslipidemia and diabetes mellitus.

**Table S38**. Multiple Analysis of Relationships between Low Brachial Intima-media Thickness and Variables

| Variables | Red blood cell  < 3.80 x 10^6^/μL | | Red blood cell  3.80-4.19 x 10^6^/μL | | Red blood cell  4.20-4.59 x 10^6^/μL | | Red blood cell  4.60-4.99 x 10^6^/μL | | Red blood cell  5.00-5.39 x 10^6^/μL | | | Red blood cell  5.40 x 10^6^/μL ≤ | |
| --- | --- | --- | --- | --- | --- | --- | --- | --- | --- | --- | --- | --- | --- |
|  | OR  (95% CI) | P value | OR  (95% CI) | P value | OR  (95% CI) | P value | OR  (95% CI) | P value | OR  (95% CI) | P value | OR  (95% CI) | | P value |
| Unadjusted model | 0.2  (0.06-0.36) | <0.01 | 0.2  (0.14-0.43) | <0.01 | 0.3  (0.21-0.53) | <0.01 | 0.6  (0.39-0.95) | 0.03 | 1 (reference) | | 0.8  (0.38-1.51) | | 0.43 |
| Model 1 | 0.4  (0.17-1.10) | 0.08 | 0.6  (0.30-1.05) | 0.07 | 0.6  (0.37-1.06) | 0.08 | 1.0  (0.61-1.62) | 0.98 | 1 (reference) | | 0.7  (0.35-1.54) | | 0.41 |
| Model 2 | 0.4  (0.15-1.01) | 0.05 | 0.5  (0.28-1.01) | 0.05 | 0.6  (0.34-1.02) | 0.06 | 1.0  (0.59-1.60) | 0.91 | 1 (reference) | | 0.8  (0.37-1.70) | | 0.55 |

Low tertile of brachial intima-media thickness indicates less than 0.30 mm. Model 1: adjusted for age. Model 2: adjusted for age, body mass index, current smoking, hypertension, dyslipidemia and diabetes mellitus.

**Supplemental Figures**

**Figure S1**


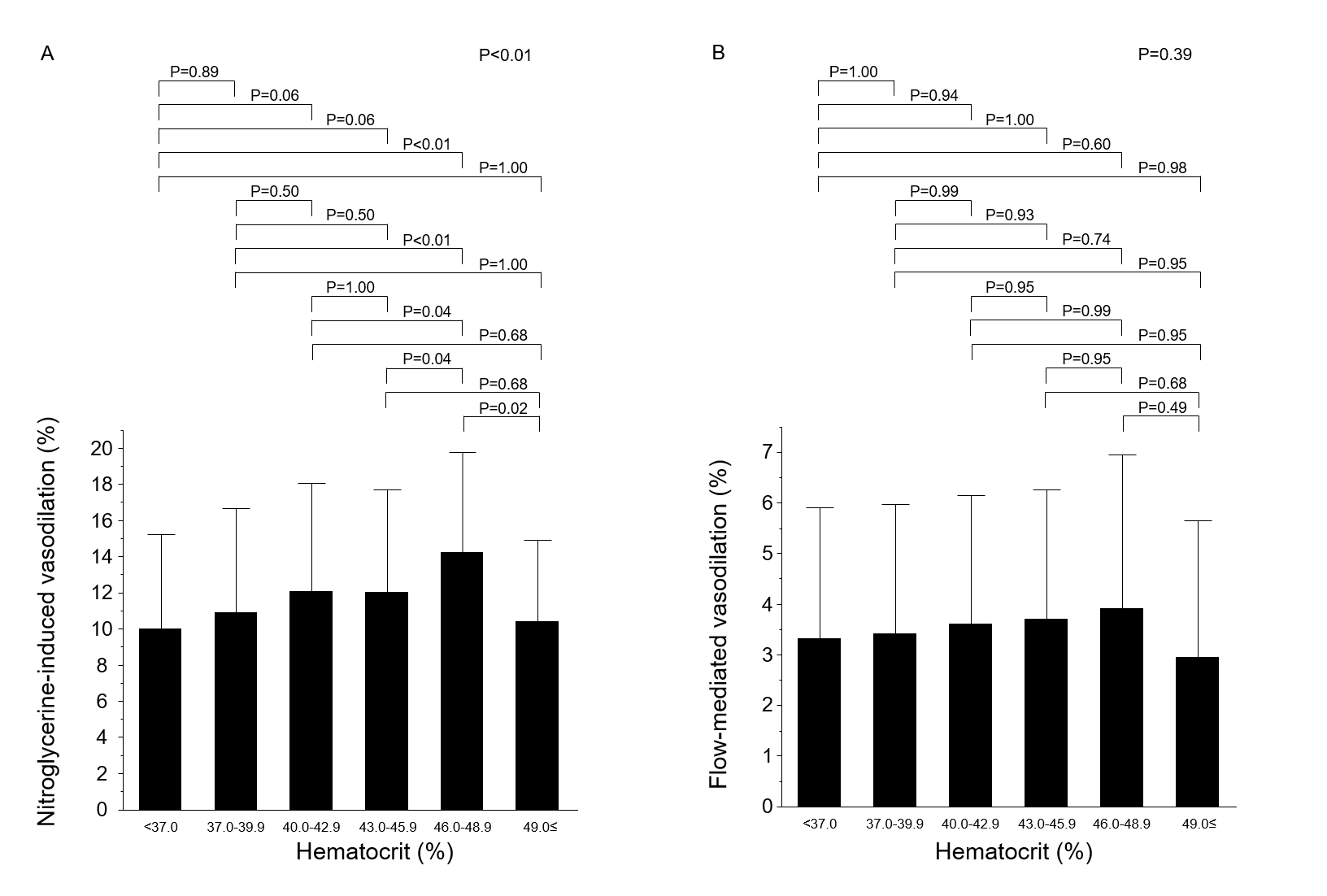


**Figure S1**. Bar graphs show nitroglycerin-induced vasodilation (A) and flow-mediated vasodilation (B) among the six groups according to hematocrit levels.

**Figure S2**.


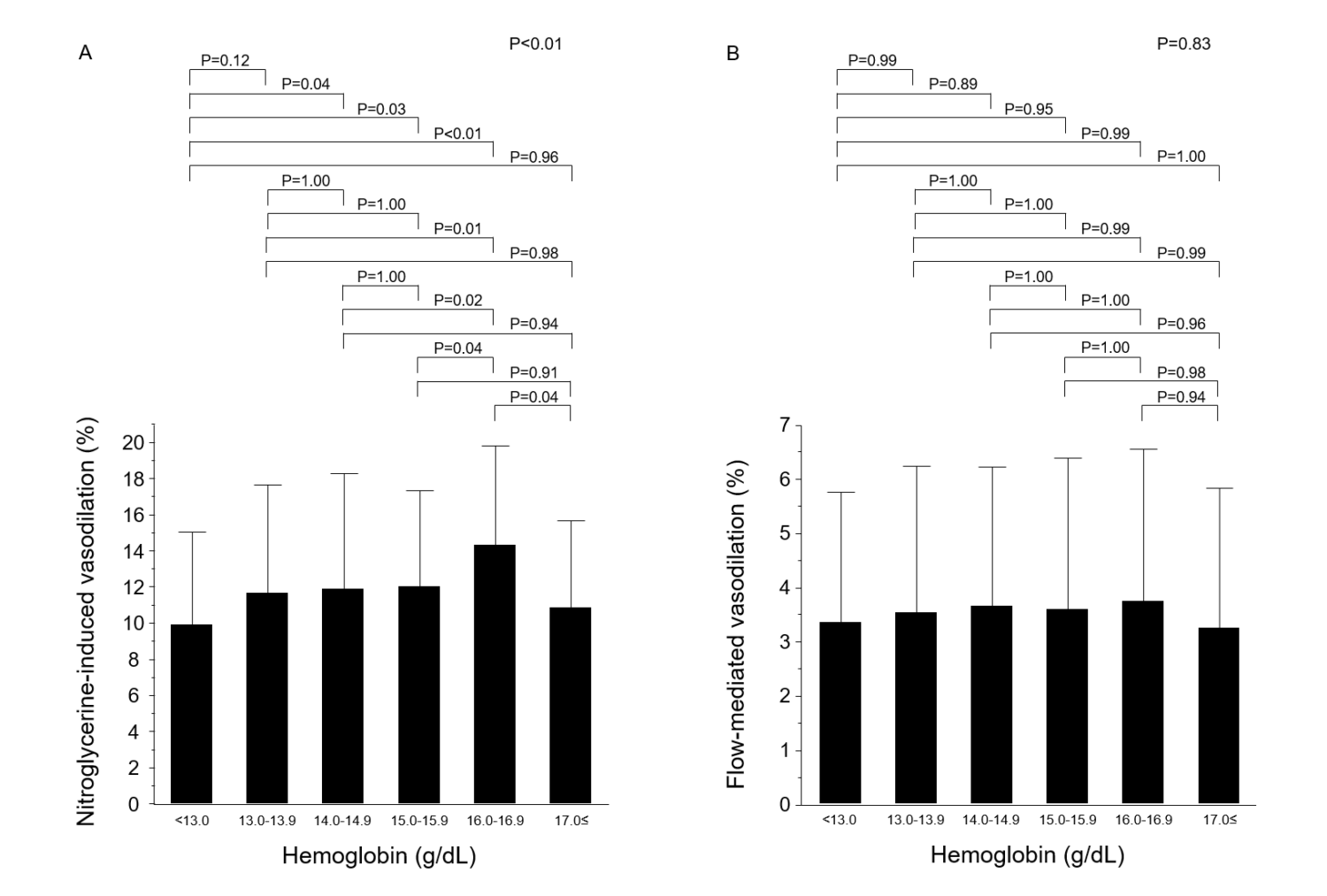


**Figure S2**. Bar graphs show nitroglycerin-induced vasodilation (A) and flow-mediated vasodilation (B) among the six groups according to hemoglobin levels.

**Figure S3.**


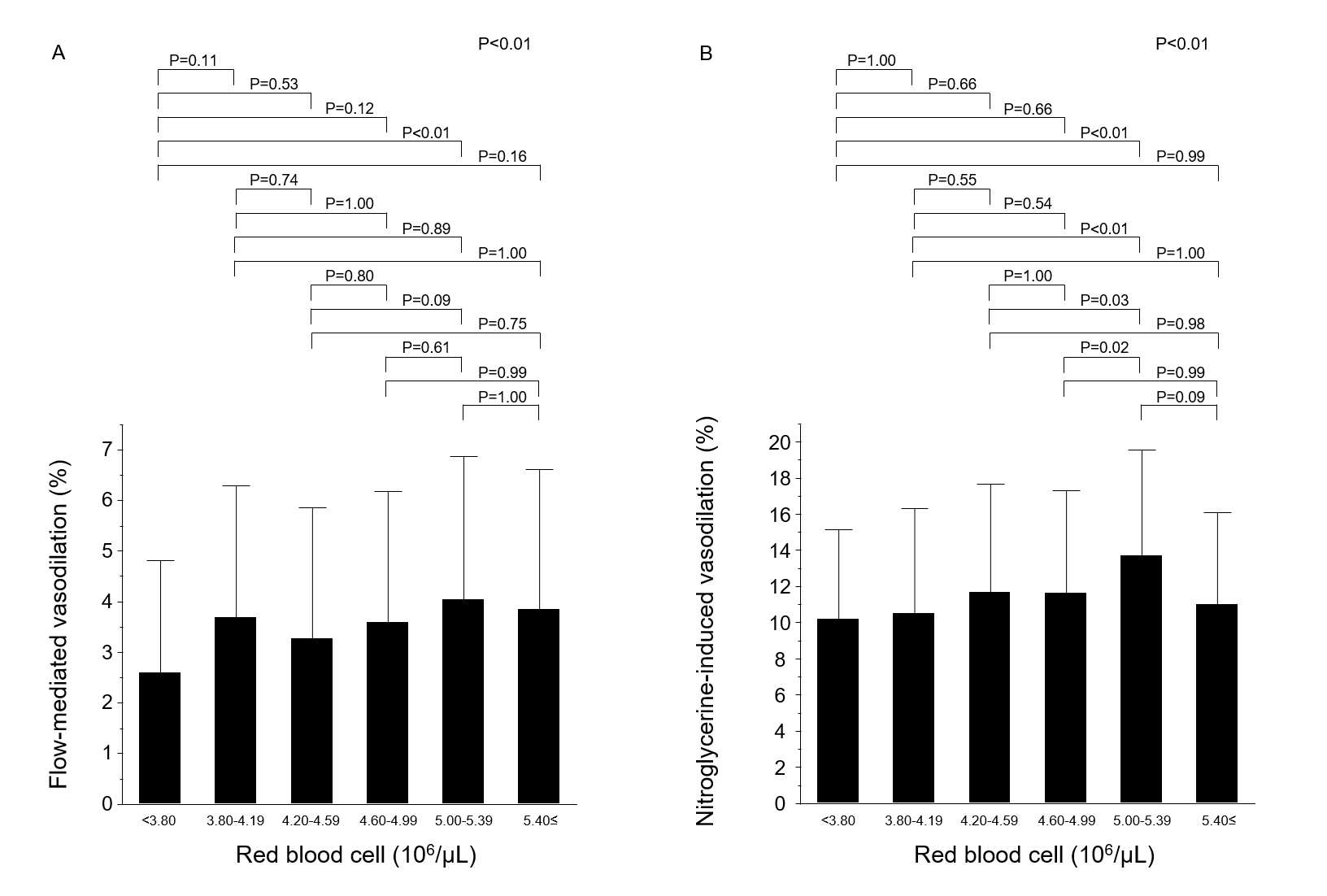


**Figure S3.** Bar graphs show flow-mediated vasodilation (A) and nitroglycerin-induced vasodilation (B) among the six groups according to red blood cell levels.

**Figure S4**.


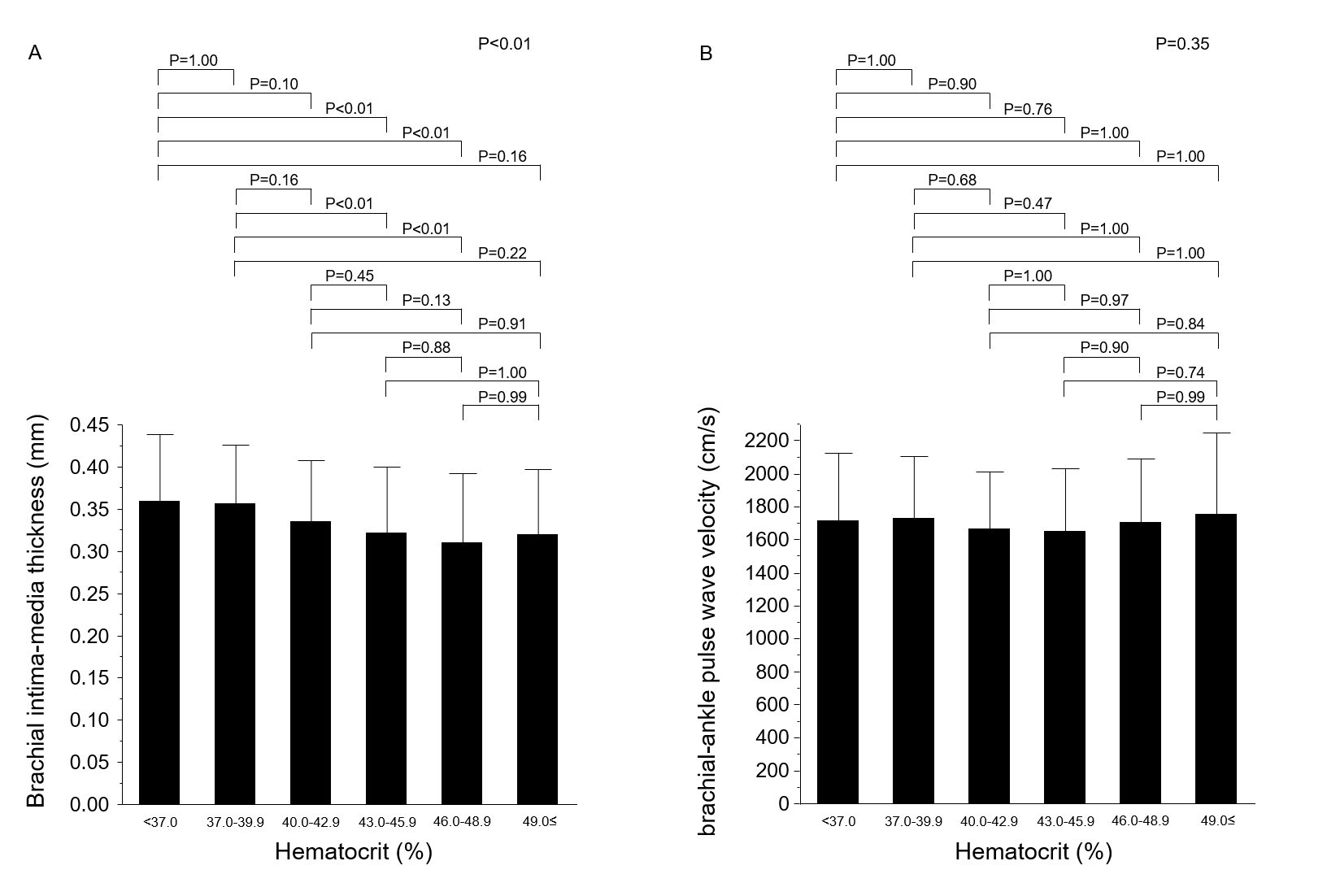


**Figure S4**. Bar graphs show brachial intima-media thickness (A) and brachial-ankle pulse wave velocity (B) among the six groups according to hematocrit levels.

**Figure S5.**


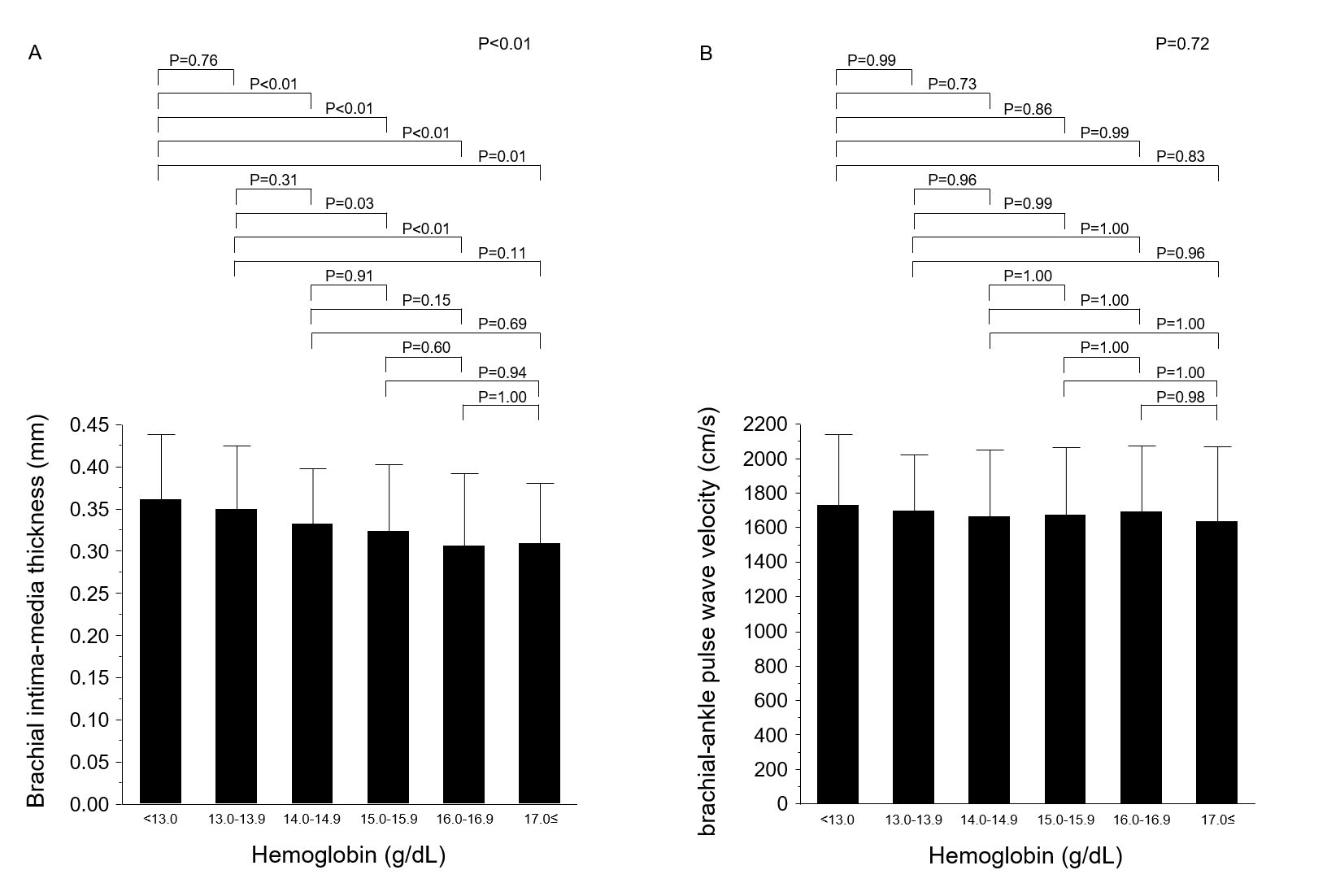


**Figure S5.** Bar graphs show brachial intima-media thickness (A) and brachial-ankle pulse wave velocity (B) among the six groups according to hemoglobin levels.

**Figure S6.**


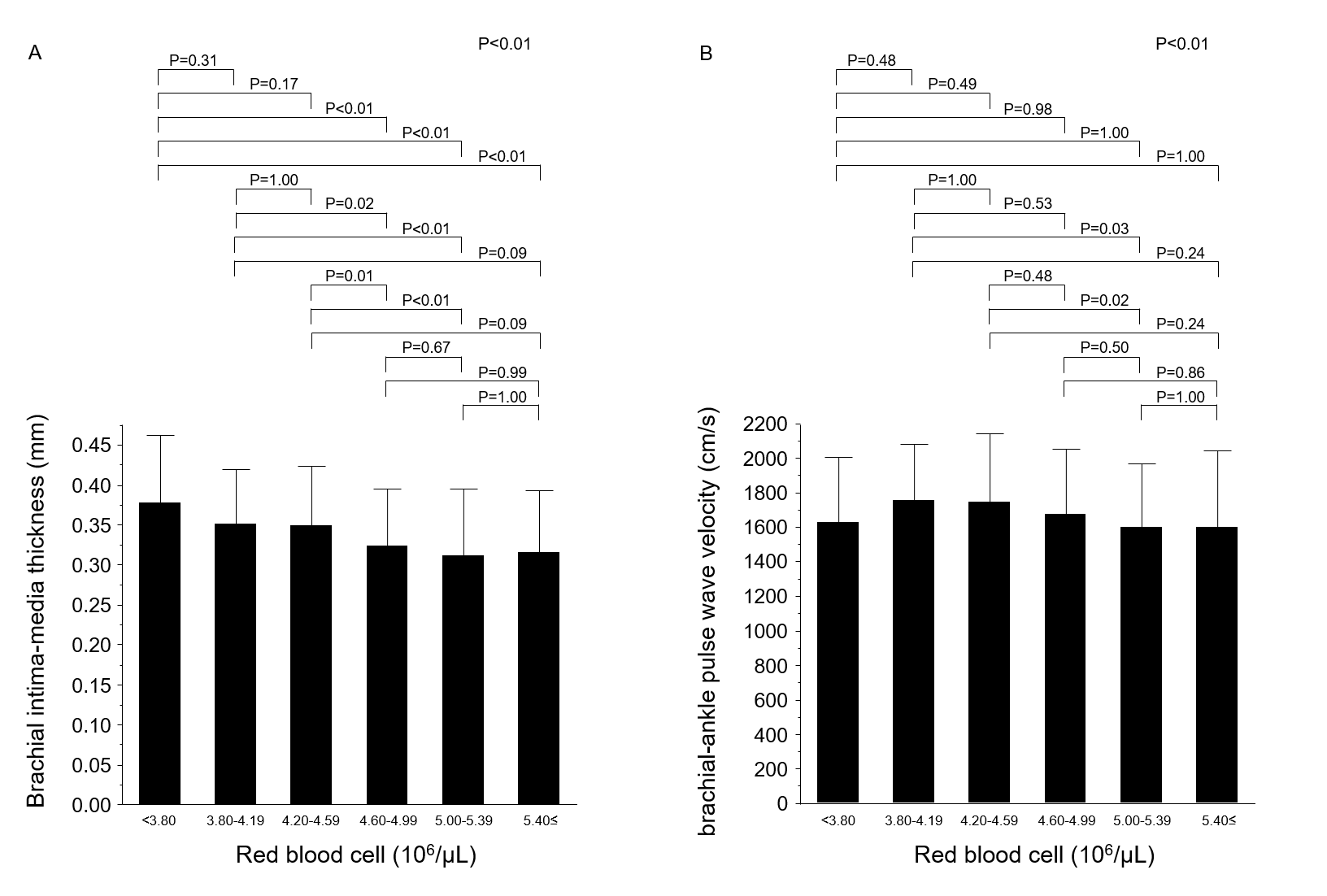


**Figure S6.** Bar graphs show brachial intima-media thickness (A) and brachial-ankle pulse wave velocity (B) among the six groups according to red blood cell levels.
